# CAFs Interacting With TAMs in Tumor Microenvironment to Enhance Tumorigenesis and Immune Evasion

**DOI:** 10.3389/fonc.2021.668349

**Published:** 2021-07-14

**Authors:** Gurcan Gunaydin

**Affiliations:** Department of Basic Oncology, Hacettepe University Cancer Institute, Ankara, Turkey

**Keywords:** cancer associated fibroblasts, monocytes, tumor associated macrophages, tumor biology, tumor immunology, tumor microenvironment, macrophage polarization, M1/M2 cells

## Abstract

Cancer associated fibroblasts (CAFs) and tumor associated macrophages (TAMs) are among the most important and abundant players of the tumor microenvironment. CAFs as well as TAMs are known to play pivotal supportive roles in tumor growth and progression. The number of CAF or TAM cells is mostly correlated with poor prognosis. Both CAFs and TAMs are in a reciprocal communication with the tumor cells in the tumor *milieu*. In addition to such interactions, CAFs and TAMs are also involved in a dynamic and reciprocal interrelationship with each other. Both CAFs and TAMs are capable of altering each other’s functions. Here, the current understanding of the distinct mechanisms about the complex interplay between CAFs and TAMs are summarized. In addition, the consequences of such a mutual relationship especially for tumor progression and tumor immune evasion are highlighted, focusing on the synergistic pleiotropic effects. CAFs and TAMs are crucial components of the tumor microenvironment; thus, they may prove to be potential therapeutic targets. A better understanding of the tri-directional interactions of CAFs, TAMs and cancer cells in terms of tumor progression will pave the way for the identification of novel theranostic cues in order to better target the crucial mechanisms of carcinogenesis.

## Introduction

It is known that the tumor microenvironment consists of several different types of cells in addition to cancer cells such as immune cells, fibroblasts as well as capillaries, basement membrane and extracellular matrix (ECM) (1–4). The dynamic and complex stroma interactions provide the conditions for tumor cell survival, growth and invasiveness. In addition to inflammatory cells, pro-inflammatory cytokines secreted from those cells are among the basic components of the tumor microenvironment (5). It is widely accepted that most neoplastic cells can only proliferate in a suitable microenvironment. Cancer cells recruit numerous cells to the tumor microenvironment and most of those cells become the *cat’s-paw* for the cancer cells; culminating in tumor survival, growth, invasion and metastasis. Cancer associated fibroblasts (CAFs) are one of the most crucial cells in the tumor *milieu*. CAFs in fact represent a heterogeneous population. The heterogeneity of CAFs might stem from their multiple origins. Similarly, tumor associated macrophages (TAMs) can also support tumor progression and increased number of TAMs is usually associated with poor outcome.

In this review; the origins, heterogeneity, activation and tumor-promoting effects of CAFs as well as tumor-supporting effects of TAMs are first outlined. Then, the interplay between CAFs and TAMs as well as their reciprocal interactions are discussed with accentuating their concerted effects on tumor progression and immune escape.

## Cancer Associated Fibroblasts

Fibroblast cells, which are one the most common cells found in connective tissues, display a branched cytoplasm that surrounds an elliptical nucleus and they express vimentin (an intermediate filament protein). They are able to produce various constituents of the ECM. Fibroblasts become activated in the tumor *milieu* and activated fibroblasts found specifically in the tumor microenvironment are defined as CAFs (6). It can be proposed that fibroblasts are among the most abundant cell types found in tumor stroma (7, 8). In fact, desmoplasia (growth of rich stroma) has long been known to be associated with tumors (9–11). Since tumors were previously depicted as “wounds that never heal” (12), CAFs resemble myofibroblasts, which are spindle shaped activated fibroblasts (13). Although CAFs can be defined as the “cells that surround cancer epithelia”, they can also be regarded as those fibroblasts which are capable of promoting tumorigenesis (8). In line with this perspective, CAF derived factors can induce a tumor supportive microenvironment as well as facilitating cancer cell metastasis (14). CAFs also play key roles in sculpturing the tumor microenvironment (15, 16). Indeed, such a role of CAFs in promoting tumor progression might also be considered to be in agreement with the original “seed and soil” hypothesis that was proposed in 1889 by Stephen Paget, who suggested that the interactions between tumor cells (*seed*) and their microenvironment (*soil*) are crucial (17, 18).

### Origin of CAFs

**Figure 1:**
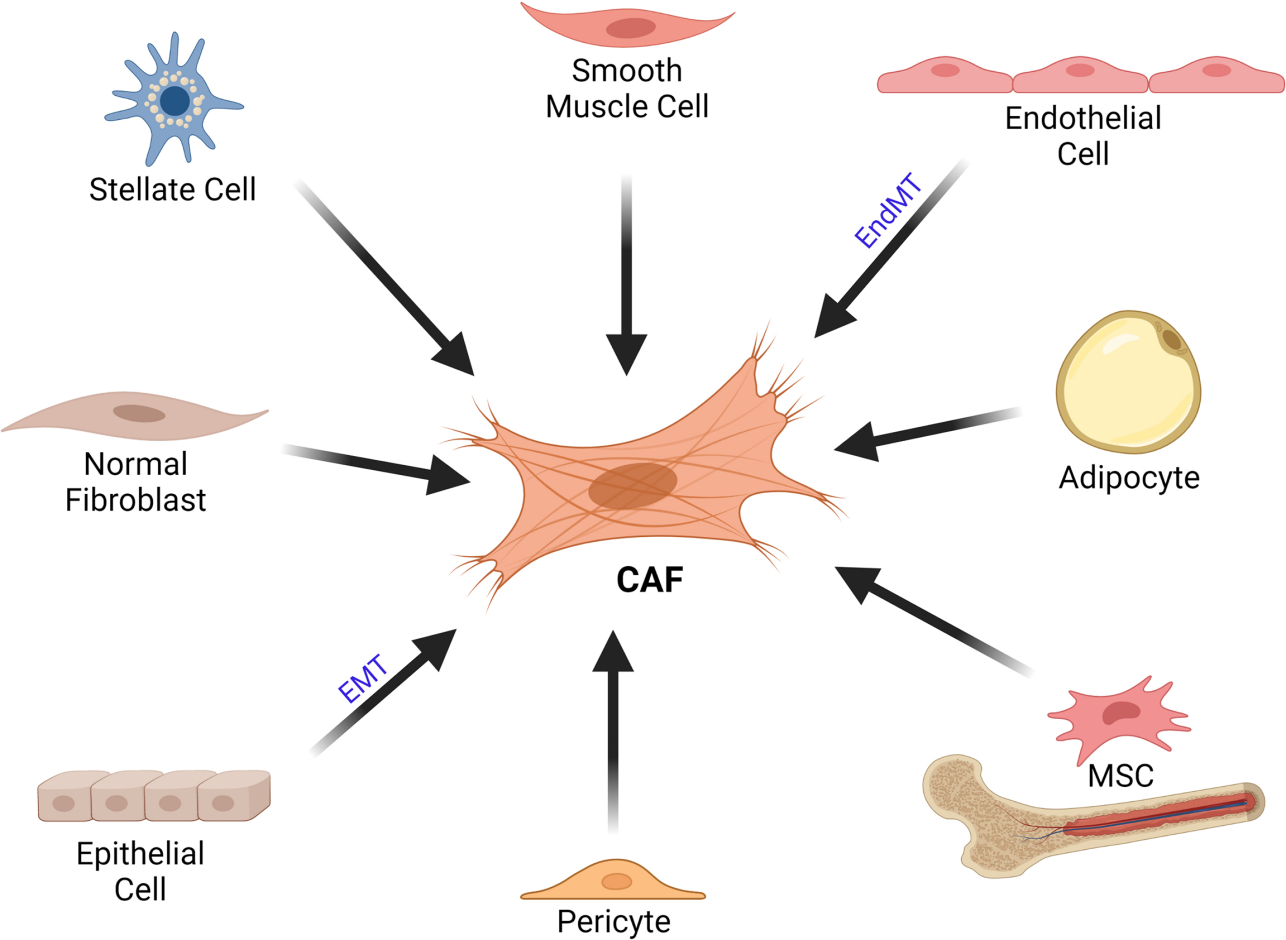
Cellular Origins of CAFs. CAFs can originate from various cell types such as stellate cells, smooth muscle cells, endothelial cells, adipocytes, MSCs, pericytes, epithelial cells as well as normal fibroblasts. CAF, cancer associated fibroblast; EMT, epithelial-mesenchymal transition; EndMT, endothelial to mesenchymal transition; MSC, mesenchymal stem cell.

Fibroblasts were initially described in the mid-1800s by Virchow and Duval as the most common cell type embedded in connective tissue in animals, demonstrating a fusiform shape (19–22). In adult tissues, Virchow described cells that produced collagen and were resistant to apoptosis (19, 23). Fibroblasts have been identified in various tissue types; however, quiescent fibroblasts do not exist in embryonic tissue (24). Thus, it remains uncertain whether the activated fibroblasts originate from mesenchymal stem cells (MSCs) or fibrocytes in adults (24). In spite of the vast literature about the features and functions of CAFs, the debate about the multiple origins for CAFs is still ongoing (**Figure 1**). The origin of CAFs can even vary depending on their location even in a single tumor and it is likely mixed (25). Most of the CAFs probably originate from mesoderm-derived precursor cells. During the tumorigenesis process, residing fibroblasts in the tissue also expand in response to injury caused by the tumor (24). In addition, CAFs can also be recruited from the bone marrow (24). Bone marrow derived MSCs can differentiate into CAFs and express α-smooth muscle actin (α-SMA) as well as fibroblast activation protein (FAP) (26). Furthermore, trans-differentiation of other cells of the tumor microenvironment may also give rise to CAF-like cells (25, 27–29). Conversion of adipocytes into CAFs has been reported in several studies (30–34). In addition, endothelial cells can also give rise to CAFs through endothelial to mesenchymal transition (EndMT) (27, 35–37). Zeisberg et al. demonstrated that transforming growth factor-β (TGF-β) 1 might promote proliferating endothelial cells to undergo phenotypic conversion into fibroblast-like cells (35). TGF-β is able to induce expressions of mesenchymal markers such as fibroblast-specific protein 1 (FSP1) and SMA; cause loss of endothelial markers such as CD31; resulting in EndMT (35). Another possible source of CAFs is pericytes (38–40). Furthermore, Wikström et al. suggested that smooth muscle cells could also give rise to CAFs (41). An interesting study by Kurashige et al. investigated the origin of CAFs in humans after sex-mismatched bone marrow transplantation (42). They reported that majority of α-SMA^+^ CAFs in liver, mammary gland and oral mucosa obtained 3–19 years after bone marrow transplantation were recipient derived cells; whereas, the peritumoral α-SMA^-^ fibroblast-like cells were mostly bone marrow derived human leukocyte antigen (HLA)-DR^+^ myeloid cells (42).

CAFs can originate *via* induction of tissue fibroblasts by tumor derived factors; in a way similar to the mechanism seen in myofibroblasts at wound healing (29). Tumor cell derived TGF-β, platelet-derived growth factor (PDGF) and fibroblast growth factor (FGF) can activate tissue fibroblasts. The mesenchymal–mesenchymal transition (MMT) results in the expression of CAF specific genes in fibroblasts such as α-SMA (43–46). Toullec et al. reported that stromal derived factor 1 (SDF-1) is an effective factor for the activation of resident fibroblasts in tumors (47). In a very recent study, Helms et al. investigated the differentiation of pancreatic stellate cells during tumor progression *in vivo* and reported that pancreatic stellate cells could give rise to a minor subset of pancreatic ductal adenocarcinoma CAFs (48). It was also suggested that miRNAs have regulatory roles in the transformation of normal fibroblasts to CAFs (49). Moreover, cells of epithelial origin are among the possible sources of CAFs and carcinomas may also contribute to the CAF population. Epithelial cells can obtain mesenchymal features and give rise to fibroblasts *via* epithelial-mesenchymal transition (EMT) (8, 50, 51), which can be induced by various factors such as PDGF, TGF-β, epidermal growth factor (EGF); resulting in the loss of E-cadherin expression (28, 52).

Finally, studies that utilize lineage tracing have also shed some light on the origin of CAFs (53, 54). Raz et al. investigated the origin of stromal subtypes. They reported that bone marrow derived mesenchymal stromal cells were recruited to primary breast tumors and lung metastases, and differentiated into CAF-like cells (55). Moreover, the expression profile of such bone marrow derived CAF-like cells was different from that of resident fibroblasts (55). In another study, Null et al. utilized *in vivo* genetic labelling of periostin^+^ subpopulations and their progeny in order to investigate their expansion/function during mammary tumor growth and metastasis (56). They proposed that highly metastatic cancer cells mobilized periostin expressing CAFs in the tumor microenvironment. Eventually, the dearth of specific markers for fibroblasts limits to some extent the broader application of the lineage tracing technique. Although we currently have a substantial understanding about the origins of CAF, the controversy concerning the importance of these mechanisms in terms of constituting the CAF population in a given tumor has not been resolved yet.

### Heterogeneity of CAFs

**Figure 2:**
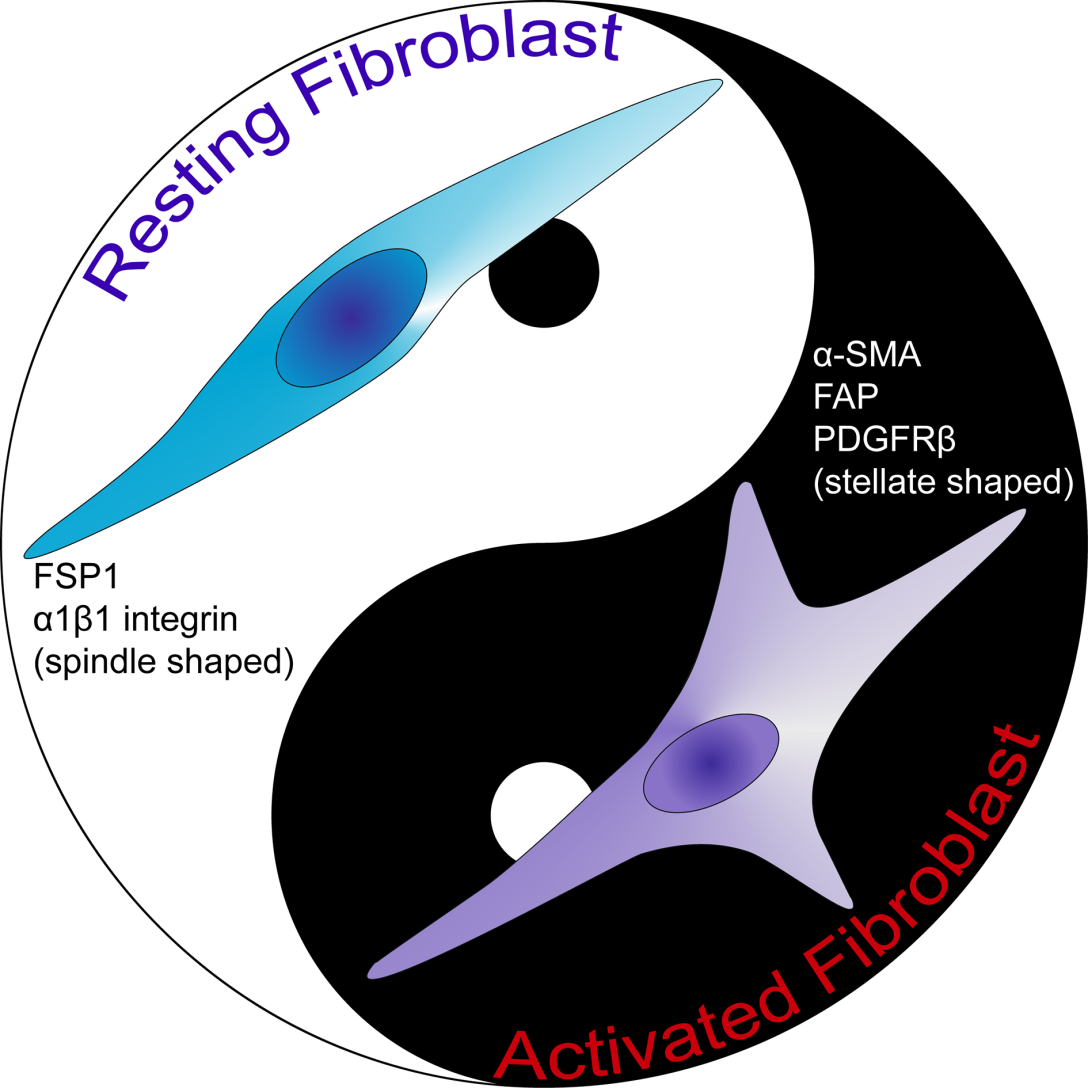
The comparison of activated fibroblast and resting fibroblast cells. Resting fibroblast cells are morphologically spindle shaped in contrast to activated fibroblast cells which are stellate shaped. Activated fibroblast cells express α-SMA, FAP and PDGFRβ. FAP, fibroblast activation protein; FSP1, fibroblast-specific protein 1; PDGFRβ, platelet-derived growth factor receptor-β; α-SMA, α-smooth muscle actin.

CAFs as well as activated fibroblasts are known to be very heterogeneous, displaying different expression patterns (**Figure 2**) (24, 57). Kalluri suggested that fibroblasts can be regarded as resting mesenchymal cells with the potential to be activated to give rise to MSCs (24). In line with this notion, it should be kept in mind that fibroblasts represent the most frequently used cell type as a source of induced pluripotent stem cells (iPSCs). In addition, fibroblast cells demonstrate a high level of plasticity and multipotency (58–61). Thus, various origins of precursor fibroblasts may explain the reason for the heterogeneity of fibroblasts. As discussed *vide supra*, activated fibroblasts can originate from various cell types (2, 8, 14, 62–66). Understanding the relative importance of each group of fibroblasts originating from different cell types will be crucial in deciphering the effects of activated fibroblasts in the disease setting. Furthermore, even the normal fibroblasts in various anatomic locations of the body can be classified as distinct cell types in light of differential gene expressions (57). As one can expect, such a heterogeneity persists also in terms of CAFs. Indeed, novel approaches such as single-cell RNA sequencing revealed significant transcriptional heterogeneity in CAF populations in murine and human studies (67–69).

In fact, CAFs are at present widely accepted to be a heterogeneous population. A recent seminal Consensus Statement on the basis of a meeting of experts in the field, underlined the degree of specialization among these cells (70). Such a specialization may explain the distinct functional sub-groups of CAFs identified by various markers (71). Our current understanding of these cells include the fact that most markers do not represent the whole CAF population. Several markers may be utilized to define CAFs such as α-SMA, FSP1, vimentin, desmin, FAP and platelet-derived growth factor receptor (PDGFR) (72). It should be kept in mind that these markers are not specific either for CAFs or for fibroblasts. Markers like FSP1 and α-SMA define diverse populations of CAFs (73). Due to the variation in terms of expressions of these markers, Sugimoto et al. described CAFs rather as a heterogeneous group of cells (7). Hence, it seems clear that more thorough classification of these cells as well as extensive analyses of functions of CAF subclasses are required in order to develop effective treatment options that target CAFs (74).

**Figure 3:**
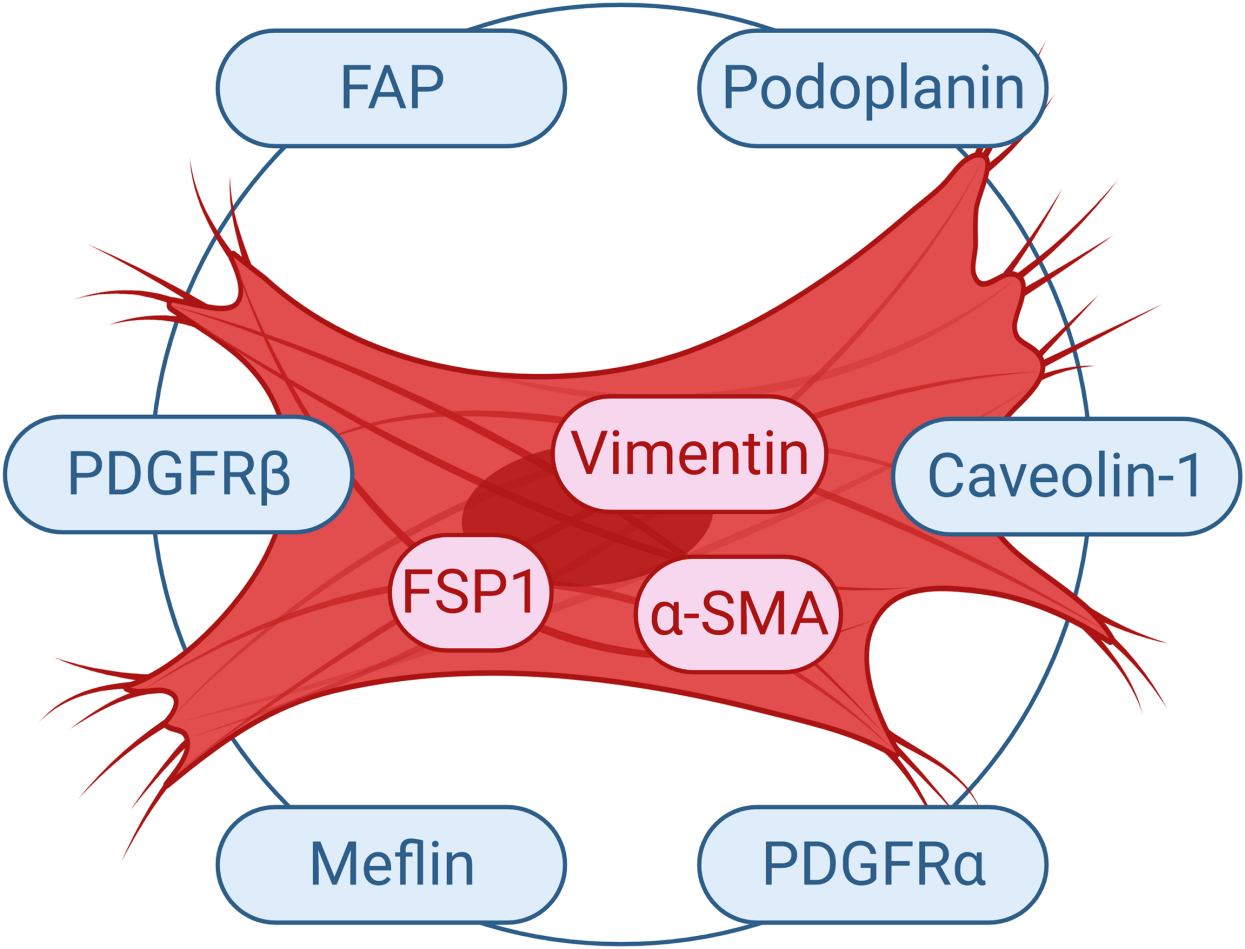
Commonly Used Markers for CAFs. Selected markers used to study CAF cells are demonstrated. FAP, fibroblast activation protein; FSP1, fibroblast-specific protein 1; PDGFR, platelet-derived growth factor receptor; α-SMA, α-smooth muscle actin.

Currently, there are no markers that specifically define CAFs. The high heterogeneity and the lack of specific markers for these cells result in poorly understanding of this population of cells in respect to their biology and subtypes (75). Nevertheless, α-SMA is generally utilized as a CAF marker (73); since the signature expression profile of these cells is still vague. The critical problem is the fact that many of the proposed markers are either not expressed by all CAF cells or not specific solely to fibroblast cells. Even though FSP1 was suggested as a specific marker for fibroblasts (76), several activated fibroblasts were reported not to express FSP1 (73). In fact, there exists a unique FSP1 expressing CAF subpopulation in the tumor microenvironment, which is distinct form α-SMA^+^ myofibroblasts (7). The role of such FSP1^+^ CAFs should be further investigated. Several other markers such as vimentin and PDGFRβ can detect both fibroblasts and myofibroblasts in the tumor microenvironment. In fact, Orimo and Weinberg proposed that CAFs include populations of both myofibroblasts and fibroblasts present within tumor (73). Although tumor stromal fibroblasts in general may express α-SMA, FAP, vimentin, neuron-glial antigen-2 (NG2) chondroitin sulfate proteoglycan, PDGFRβ, prolyl 4-hydroxylase and FSP1; α-SMA and FAP can be used to discriminate myofibroblasts form fibroblasts in the tumor tissue (**Figure 3**) (73).

### Activation of Fibroblasts

**Figure 4:**
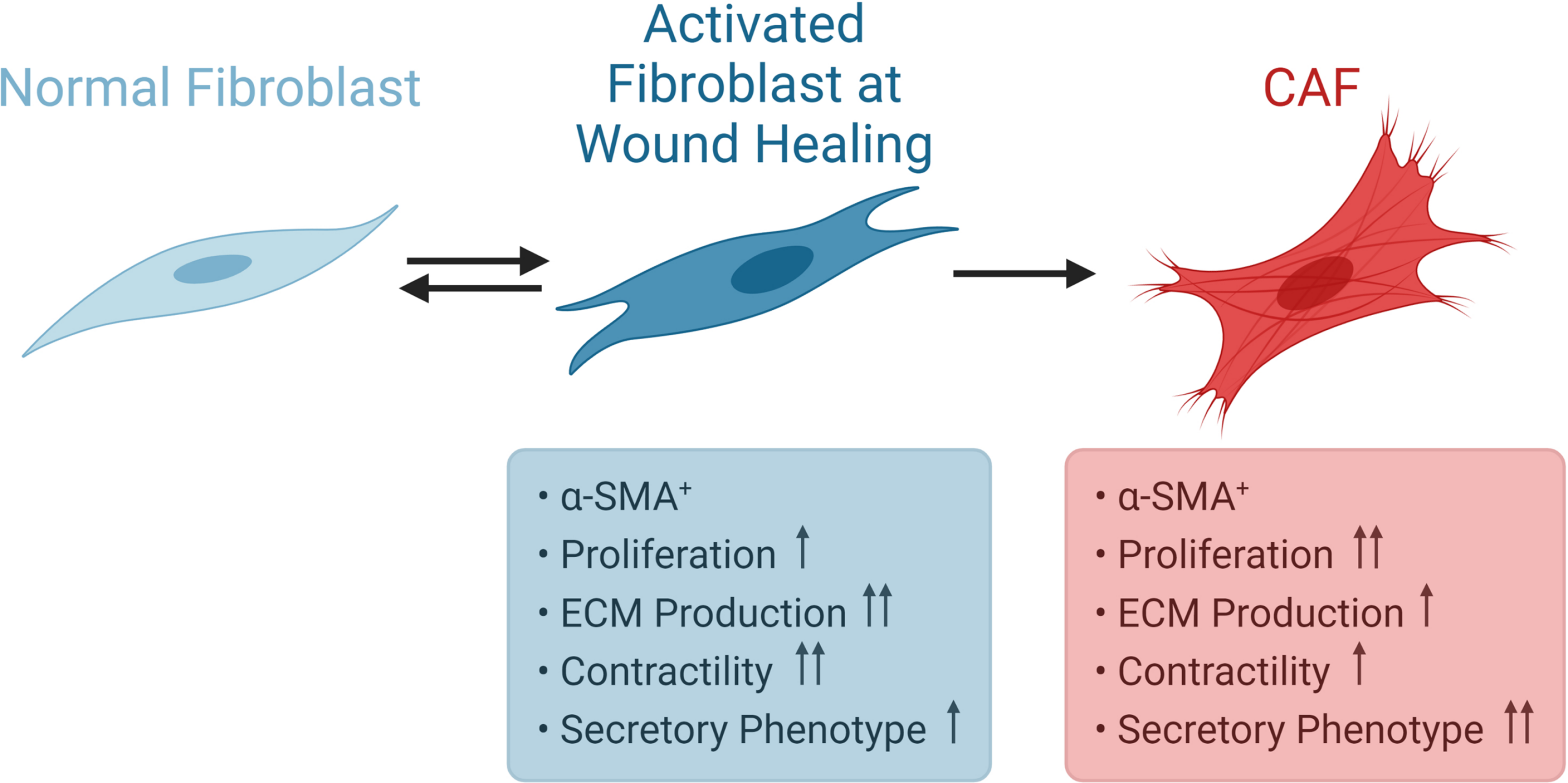
Activation of Fibroblasts. Normal fibroblasts are spindle shaped cells. During wound healing, fibroblasts become reversibly activated and express α-SMA. CAFs also express α-SMA, but display higher proliferative capacity and secretory phenotype. CAF, cancer associated fibroblast; α-SMA, α-smooth muscle actin.

Resting fibroblast cells in tissues are spindle shaped single cells. In case of tissue injury, they become reversibly activated in order to take part in tissue repair (77). Activated fibroblasts turn into myofibroblasts; since they acquire contractile stress fibers, express α-SMA (78). When the wound healing process is completed, the activation of fibroblasts is reversed by either reprogramming or removal from the granulation tissue by a specific type of programmed cell death called nemosis (24, 79, 80). Activated fibroblasts are known to gain secretory functions such as production of higher amounts of ECM and also demonstrate increased proliferation (81). Such an increased bioactivity is called as fibroblast activation (81). In situations of prolonged injurious stimuli, such as fibrosis or cancer, they gain further proliferative and secretory functions (**Figure 4**). Kalluri suggested that such fibrosis and cancer associated fibroblasts, respectively, bear epigenetic regulations which may result in irreversible activation (24, 82). For instance, resident fibroblasts become activated and myofibroblasts are generated from bone marrow derived cells, pericytes and endothelial cells in obstructive nephropathy (83). CAFs are similar to normal activated fibroblasts in terms of expressing α-SMA, but demonstrate higher proliferative capacity and secretory phenotype. On the other hand, the perspective proposing that CAFs include populations of both myofibroblasts and fibroblasts should also be kept in mind (73).

### The Role of CAFs on Tumor Progression

Several aspects of multi-step carcinogenesis have been thoroughly investigated over the last decades. Identification of key oncogenes and tumor suppressor genes have led to the establishment of carcinogenesis models that incorporate gain or loss of various gene functions. Although such information is truly priceless, this seed-centric model does not sufficiently underline the importance of the microenvironment, “soil”, which the tumor cells reside in. Cancer cells cannot be regarded as isolated entities. They actively interact with their *milieu*, which contains several different types of cells including fibroblasts (84). The highly complex tumor microenvironment greatly affects tumor progression (85). Given the fact that Paget proposed the “Seed and Soil” hypothesis more than a century ago (17), this concept is in fact cannot be regarded as new.

The normal histological organization of most epithelial cells provides a way of separation from the stroma by a basement membrane, which contains stromal cells such as normal fibroblasts. In fact, tumor stroma seems to exert a bimodal influence on cancer development. It inhibits tumor growth in normal tissue, whereas it may facilitate tumor growth and migration during tumor progression (86). During the transition from normal to neoplastic epithelial cells, there exists a reciprocal and paracrine crosstalk between the epithelium and the stroma, which results in activation of members of the stroma such as fibroblasts and maturation of blood vessels. This, in turn, potentiates the development of cancer (86). In line with this reciprocal interaction, tumor progression is characterized with further epithelial proliferation and activation of stromal cells. In fact, CAFs may also be regarded as components of the host’s response to injury caused by the growing tumor (24, 87). Thus, CAFs may demonstrate anti-tumor responses during the early stages of neoplasia (24, 88). It is likely that fibroblasts become activated at early stages of carcinogenesis and give rise to CAFs, which take active part in tissue remodeling (89). The anti-tumor effects of CAFs at initial stages of neoplasia may be replaced by pro-tumoral effects as the tumor grows. CAF derived molecules can be implicated in cancer cell survival and proliferation under such circumstances. LeBleu and Kalluri suggested that pro-tumoral effects of CAFs might evolve gradually (89). In addition to several inherent changes in tumor cells, they also alter their microenvironment *via* secretory molecules such as growth factors, interleukins, colony stimulating factors. As a result, several mechanisms including the induction of angiogenesis (90, 91), inflammatory cell recruitment (92), immune modulation (93, 94) and ECM remodeling (95) facilitate tumor progression. In addition, CAF derived factors can promote therapy resistance and immune exclusion (96, 97). In an autochthonous model, Feig et al. demonstrated that immune control of pancreatic ductal adenocarcinoma growth could be achieved *via* depleting FAP^+^ CAFs (98). This depletion uncovered the anti-tumor effects of immunological checkpoint antagonists [α-cytotoxic T-lymphocyte associated protein 4 (CTLA-4) and α-programmed cell death 1 ligand 1 (PD-L1)]. The researchers also suggested that CXCL12 (SDF-1) might account for the overriding immunosuppression by the FAP^+^ cells (98).

Activated fibroblasts may even directly facilitate metastasis (86, 99, 100). Peña et al. reported that stanniocalcin-1 expression by CAFs could drive metastasis of colorectal cancer (101). Furthermore, tumors formed in the presence of stanniocalcin-1 deficient fibroblasts demonstrated fewer and smaller distant metastases in an orthotopic mouse model of colorectal cancer (101). In an interesting study, Pelon et al. described four CAF subpopulations in metastatic lymph nodes from breast cancer samples. They reported that CAF heterogeneity in axillary lymph nodes promotes metastases in breast cancer *via* complementary mechanisms (102). Studies utilizing single-cell RNA sequencing can provide important data about CAF functions in early, advanced and metastatic tumors (103–105). Our current knowledge concerning the CAFs at metastatic sites is very limited. Shani et al. recently investigated the transcriptional co-evolution of lung fibroblasts isolated from transgenic mice at defined stage-specific time points of metastases formation. They suggested that fibroblasts in lung metastases were transcriptionally plastic, showing stage-specific gene signatures (106). Indeed, it has long been known that cancer cells can migrate from the primary site with CAFs (107). In a mouse model, Duda et al. demonstrated that the viability of circulating metastatic cancer cells was higher if they were incorporated in tumor-stroma cell fragments (107). A seminal study by Kalluri et al. demonstrated the important role of local fibroblasts in providing the suitable microenvironment for metastatic colonization (108). VEGF-A and Tenascin-C produced by specific stromal cells were found to be important for metastatic colonization (108). In another study, Benedicto et al. reported that disruption of CXCR4/CXCL12 axis by CXCR4 antagonist *AMD3100* inhibited the contribution of cancer and stromal cells to the metastatic cascade in the liver (109).

Our current understanding of the effects of tumor stroma on tumor progression and the course of metastatic events is rapidly expanding (110, 111). Cancer cells recruit different cell types which in turn help support tumor growth and progression (110). CAFs exert a myriad of effects on tumor progression. They were shown to directly facilitate tumor growth in several studies. In an important study, Weinberg et al. demonstrated that CAFs facilitated breast cancer progression more potently in a co-implantation tumor xenograft model, when compared with their normal counterparts (112). CAFs are also able to affect cancer cell invasion as well as metastasis (113). In an interesting study, Shapcott et al. utilized a deep learning cell identification algorithm on colon cancer diagnostic images in TCGA. They reported that increased fibroblast numbers were associated with invasion, metastasis and residual tumor (114). In a study by Gaggioli et al., fibroblast cells were shown to act as leading cells triggering proteolytic and structural modifications of the ECM to create tracks which carcinoma cells move within behind the fibroblasts, in order to achieve cell invasion into ECM (115). In a recent study, Wei et al. reported that periostin^+^ CAFs promoted lymph node metastasis (116). Periostin is an ECM protein produced by fibroblasts (117). It was previously shown to increase the proliferation and invasiveness of tumor cells (118). Wei et al. demonstrated that periostin^+^ CAFs affected lymphatic endothelial barriers in cervical squamous cell carcinoma *via* activating the integrin-FAK/Src-VE-cadherin signaling pathway in lymphatic endothelial cells, resulting in increased metastatic dissemination (116).

Tumor cells are well known to utilize exosomes to change the functions of other cells in the tumor microenvironment (119). In recent years, CAFs were also shown to take part in exosome mediated communications (120). CAF derived vesicles are able to promote the migration and invasion of cancer cells (121, 122). Qin et al. demonstrated that CAF derived exosomal miR-196a contributes to cisplatin resistance in head and neck cancer by targeting CDKN1B and ING5 (123). In another study, Wang et al. showed that CAF derived exosomes contain decreased miR-3188 levels compared to NFs. They also reported that loss of miR-3188 in exosomes contributed to malignant phenotypes of head and neck cancer cells (124). Such findings are not limited to head and neck cancer.

**Figure 5:**
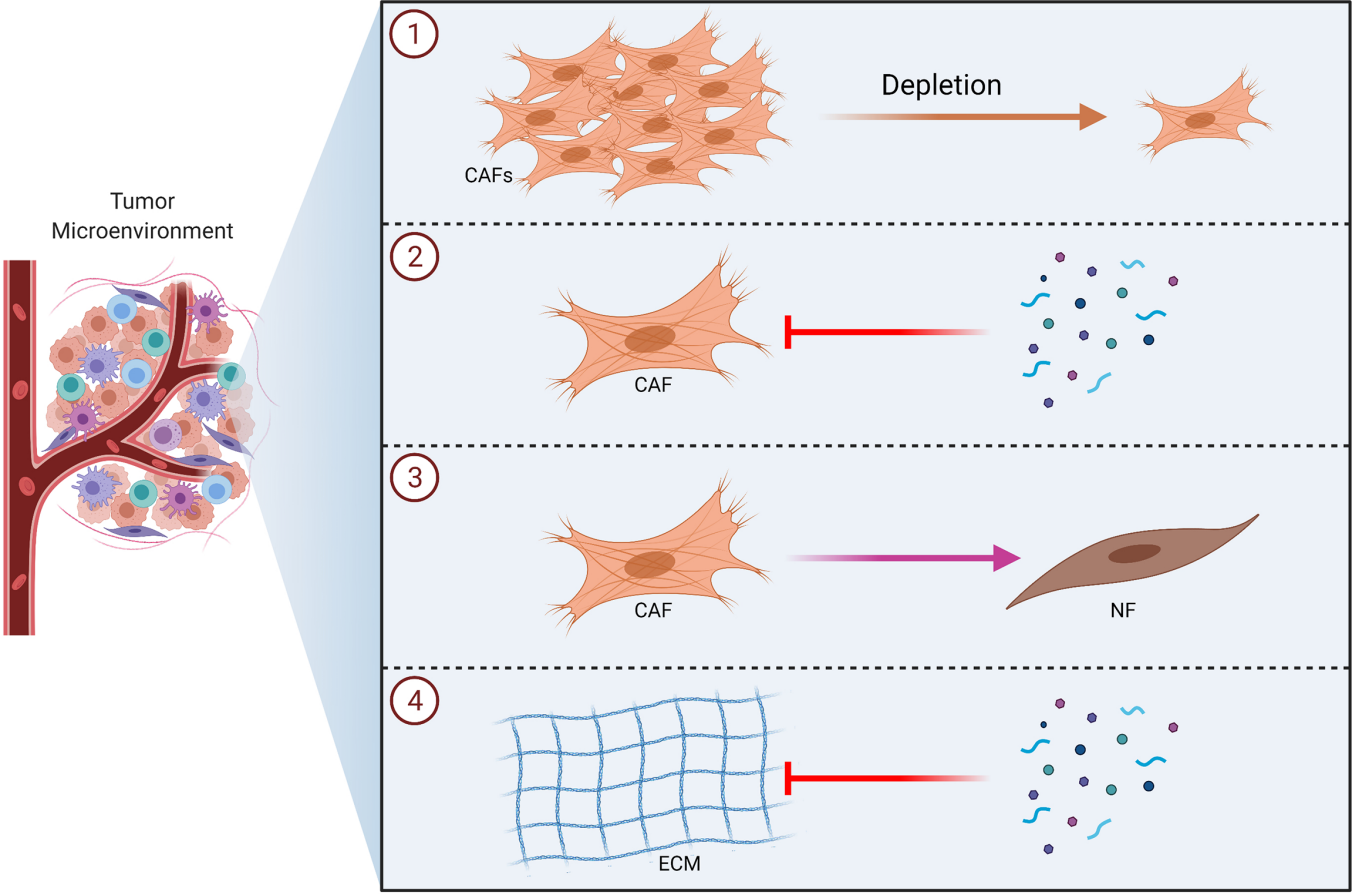
Therapeutic Strategies to Target CAFs in Tumors. Schematic illustrations of the CAF-targeting approaches including depletion of CAFs ①, blocking CAF functions ②, altering CAF activation status ③ and targeting ECM ④ are shown. CAF, cancer associated fibroblast; ECM, extracellular matrix; NF, normal fibroblast.

Various tumor derived factors can be responsible from the activation of CAFs, which may in turn support tumor growth. Cirri and Chiarugi proposed a model for the interaction of CAFs with tumor cells, which suggests that there exists a positive and reciprocal feedback between CAFs and cancer cells (82). Cancer cells induce fibroblast activation *via* factors like TGF-β; which in turn causes tumor survival, ECM remodeling, angiogenesis and EMT by secreting cytokines and growth factors (125–127). Tumor microenvironment also plays a crucial role in inducing drug resistance (128, 129). CAFs may support cancer cells in surviving from cancer drugs (130). Wang et al. reported that fibroblasts play an important role in lung cancer resistance to epidermal growth factor receptor (EGFR) tyrosine kinase inhibitors *via* hepatocyte growth factor (HGF) (131). In another study, it was found that fibroblasts might cause resistance of cancer cells to gemcitabine (132). Nakasone et al. demonstrated that the microenvironment could be implicated in drug resistance by regulating vascular permeability and immune cell infiltration (133). Such studies showing the role of CAFs in terms of drug resistance underline the importance of therapeutic approaches that will also incorporate strategies to target the tumor microenvironment in order to overcome drug resistance. Therefore, therapeutic approaches that incorporate anti-CAF strategies have been extensively studied over the recent years (134–136). Such strategies may involve depletion of CAFs, blocking CAF functions, altering CAF activation status and targeting ECM (**Figure 5**) (134, 137). In fact, several anti-CAF strategies have been investigated in the clinical setting (70).

It should also be taken into consideration that organ specificity might play a major role in terms of the effects of CAFs on tumor progression. The stromal content of solid tumors can considerably differ and it may constitute 60–90% of the total tumor mass in several tumor types (*e.g.* breast, colon, stomach, pancreatic cancer) (1, 12, 138, 139). Moreover, CAFs are extremely influential in desmoplastic reactions and they may help to create a desmoplastic tumor microenvironment (140, 141). Desmoplasia is a prominent feature of the pancreatic ductal adenocarcinoma (142). Indeed, pancreatic ductal adenocarcinoma is characterized by an extensive deposition of ECM, which is probably more than any other type of malignancy (96). Both primary tumors and metastases of pancreatic ductal adenocarcinoma are characterized by increased levels of desmoplasia (143). Desmoplasia can be regarded to promote tumor invasion in pancreas, breast, lung, esophagus, stomach and prostate (144–150). On the other hand, the effect of desmoplasia in colorectal carcinoma progression is not very clear (151). Nonetheless, desmoplastic reaction seems to be an independent prognostic factor also for colorectal cancer (152–154). Maturity of CAFs and desmoplastic reactions were reported to be associated with cancer invasion in patients with colorectal cancer (155). Given the differences in various tissues concerning the presence and/or extent of desmoplasia, CAFs may also demonstrate tissue/organ and tumor type specific alterations. Indeed, it is very difficult to determine markers in order to label CAF subpopulations that take part in tumor progression in different organs (88), and to cap it off, it is currently unclear whether specific CAF populations are preserved across tissues (70). CAF subpopulations may also demonstrate differences in early or late stage primary tumors (156). Friedman et al. reported that CAF repertoire changes over time in breast cancer progression (157). As one might expect, it is not readily possible to investigate the alterations in CAF populations in human tissue samples during disease progression, since longitudinal analyses of the same lesion is very difficult. Future studies in murine models with temporal resolution may provide priceless information concerning the alterations in CAFs during tumor progression. Furthermore, there is very little information concerning the CAF composition in metastasis sites. Novel studies suggest the presence of resembling CAF populations in different malignancy types (96). Ikenaga et al. had previously reported that CD10^+^ pancreatic stellate cells enhanced the progression of pancreatic cancer cells (158). In a more recent study, CD10 and GPR77 were also reported to define a CAF population correlated with poor survival and chemoresistance in multiple cohorts of breast and lung cancer patients (159). Numerous studies showed that different types of malignancies contain both myofibroblasts and non-myofibroblasts (38, 160–162). In contrast, several other studies have demonstrated the existence of distinct CAF populations in various cancer types (96). The kinetics of CAF functions can vary in different malignancy types, since resident fibroblasts display different organ specific transcriptomic profiles (163). Rinn et al. investigated genome wide gene expression profiles of primary fibroblasts from distinct anatomical sites and demonstrated that differences in gene expression programs were related with anatomic divisions (*i.e.* anterior-posterior, proximal-distal, dermal-nondermal) (163). In fact, even different tumor subtypes may contain diverse fibroblast populations. Tchou et al. reported that gene expression in human breast CAFs significantly varied between breast cancer subtypes (ER^+^, Her2^+^ and triple-negative breast cancer) (164). Different subpopulations of CAFs may demonstrate distinct functions even within a tumor type (165). In light of these findings, investigation of differences and similarities between CAF subpopulations in different malignancy types may pave the way for innovative strategies to precisely target CAFs.

## Tumor Associated Macrophages

Macrophages were first discovered by Ilya Mechnikov late in the 19th century (166). Mechnikov, who was awarded the 1908 Nobel Prize in Physiology or Medicine jointly with Paul Ehrlich in recognition of their work on immunity (167), championed the theory of phagocytes and described mobile cells battling invading pathogens (168). Indeed, macrophages are among the most important and abundant players of the tumor microenvironment. They are capable of affecting tumor progression, angiogenesis and therapy resistance (169–171). TAMs display a wide diversity and plasticity. TAMs were originally considered to originate from circulating monocyte precursors released from the bone marrow (172). It has also been demonstrated that many tissues contain embryonic derived populations of resident macrophages (173, 174). The exact causes of the diversity of TAMs are not clear; however, plasticity of TAMs and/or independent specific lineages generating multiple populations might account for such a heterogeneity (175). Indeed, deciphering the origin of TAMs might improve cancer immunotherapy strategies (176).

TAMs can demonstrate supportive and inhibitory effects on cancer, depending on several factors such as stage and microbiota (177). TAMs may also function as a barrier for anti-tumor immunity (178). More than 70% of human breast cancers express colony stimulating factor 1 (CSF-1), which is a key regulator of mononuclear phagocytic lineage; and expression of CSF-1 is correlated with poor prognosis (179). Macrophages are known to be highly plastic cells. They can be classically or alternatively activated. Classically activated anti-tumor macrophages (M1) and alternatively activated tumor promoting macrophages (M2) reflect the nomenclature of polarized immune responses (180, 181). In a seminal study, Hill et al. suggested that M1 or M2 dominant macrophage responses might influence the types of immune responses (182). In fact, Mantovani et al. suggested that M1 and M2 macrophages represent the extremes of a continuum of activation states. Therefore, the adaptability of macrophages in response to various conditions goes beyond such a dichotomy (181).

### Polarization of Macrophages

**Figure 6:**
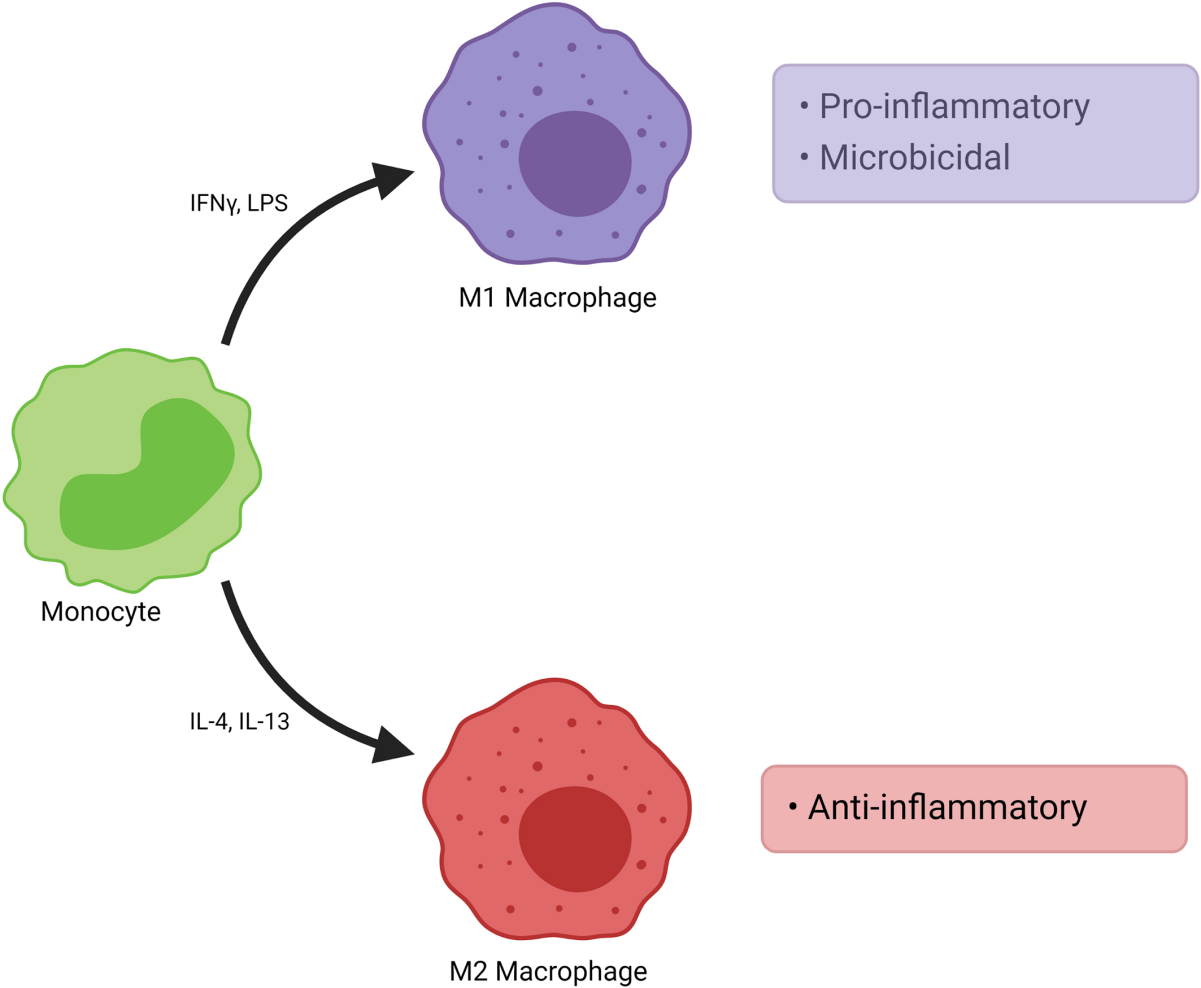
Polarization of Macrophages. Monocytes from blood differentiate into functionally distinct populations. M1 macrophages are induced by various stimuli such as IFNγ. They demonstrate pro-inflammatory and microbicidal effects. M2 macrophages are induced by stimuli such as IL-4, IL-13 and they show anti-inflammatory effects. M2 macrophages may be further divided into subpopulations such as M2a, M2b and M2c. IFNγ, interferon gamma; IL, interleukin; LPS, lipopolysaccharide.

**Figure 7:**
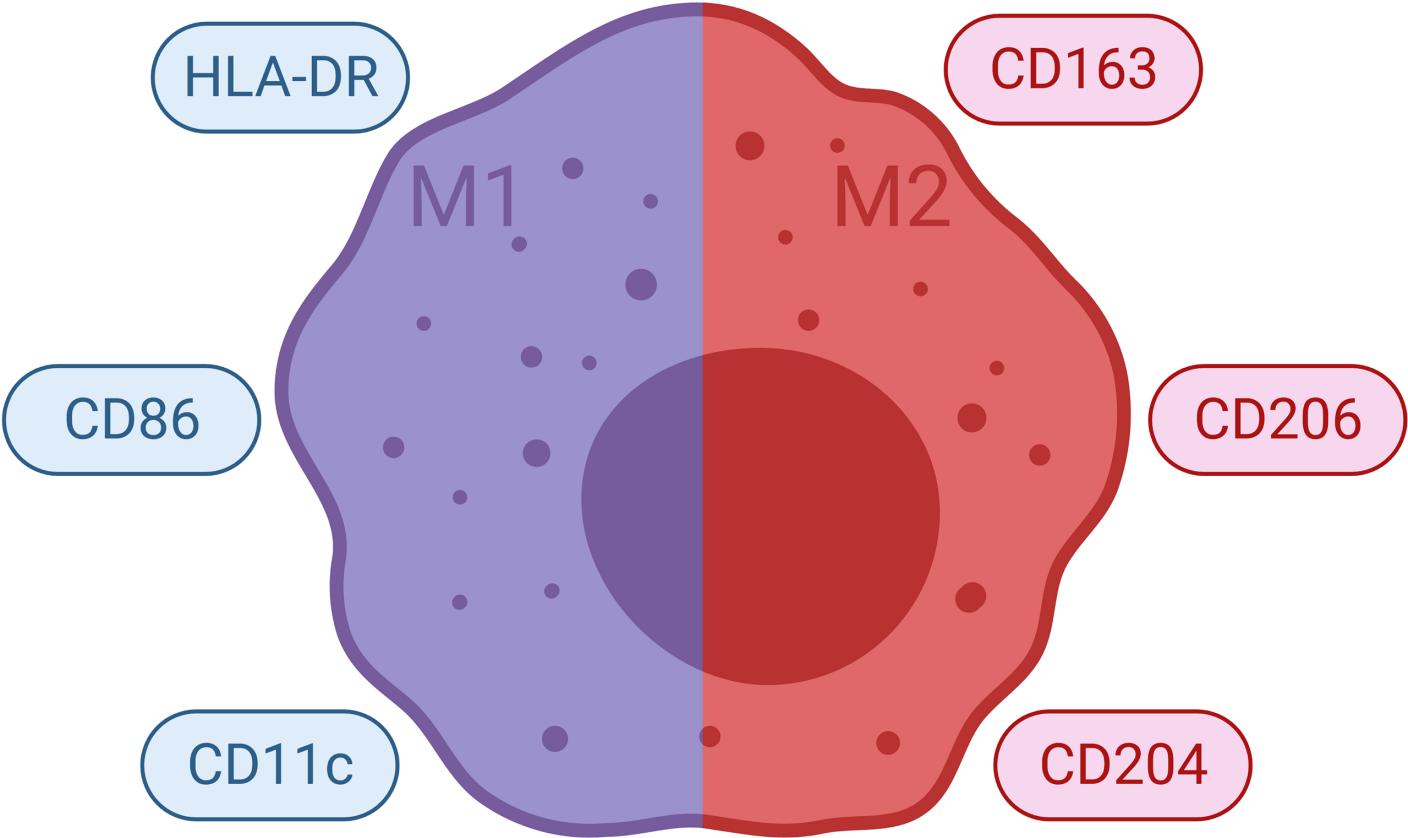
Commonly Used Markers for Macrophages. Selected markers used to distinguish macrophage phenotypes are demonstrated. HLA, human leukocyte antigen.

Monocytes from blood differentiate to M1 or M2 subtype macrophages, depending on their interactions with other cells present in tissues (**Figures 6** and **7**). Such differentiation is dependent on the environmental stimuli these cells are subject to (183–190). The first type of antimicrobial macrophage activation became known as M1 macrophages (191). In an important study in 1992, Stein et al. reported that expression of macrophage mannose receptor was inhibited by interferon gamma (IFNγ); whereas, recombinant murine IL-4 significantly enhanced macrophage mannose receptor surface expression and activity (192). IL-4 caused inflammatory macrophages to show an alternative activation phenotype, unlike that induced by IFNγ (192). Such findings paved the way for the concept of alternatively activated macrophages (M2). High IFNγ levels result in M1 differentiation, whereas high levels of interleukin (IL)-4 and IL-13 polarize macrophages into M2 subtype (180). M1 macrophages produce several pro-inflammatory mediators such as tumor necrosis factor (TNF)-α, IL-1, IL-6; as well as microbicidal and tumoricidal molecules such as reactive nitrogen/oxygen intermediates (191). On the contrary, M2 macrophages express molecules which can be involved in parasite infestation, tissue remodeling and tumor progression such as resistin-like-α, Arginase 1, chitinase-like molecules, IL-10 and mannose receptor C-type 1 (Mrc1) (193). Human M1 macrophages show high expressions of CD14, CD16, CD64, CD86, and HLA-DRα. On the other hand, M2 macrophages express CD163 and CD206 (also known as Mrc1) (183, 194–196). Nevertheless, recent studies suggest that classification of macrophages is not dichotomous and different macrophage subtypes might demonstrate shared features (197). Indeed, macrophages can constitute nearly half of the tumor mass in breast cancer. In addition, it has been shown that there is a correlation between the number of TAMs and poor prognosis (198, 199).

### The Role of TAMs on Tumor Progression

**Figure 8:**
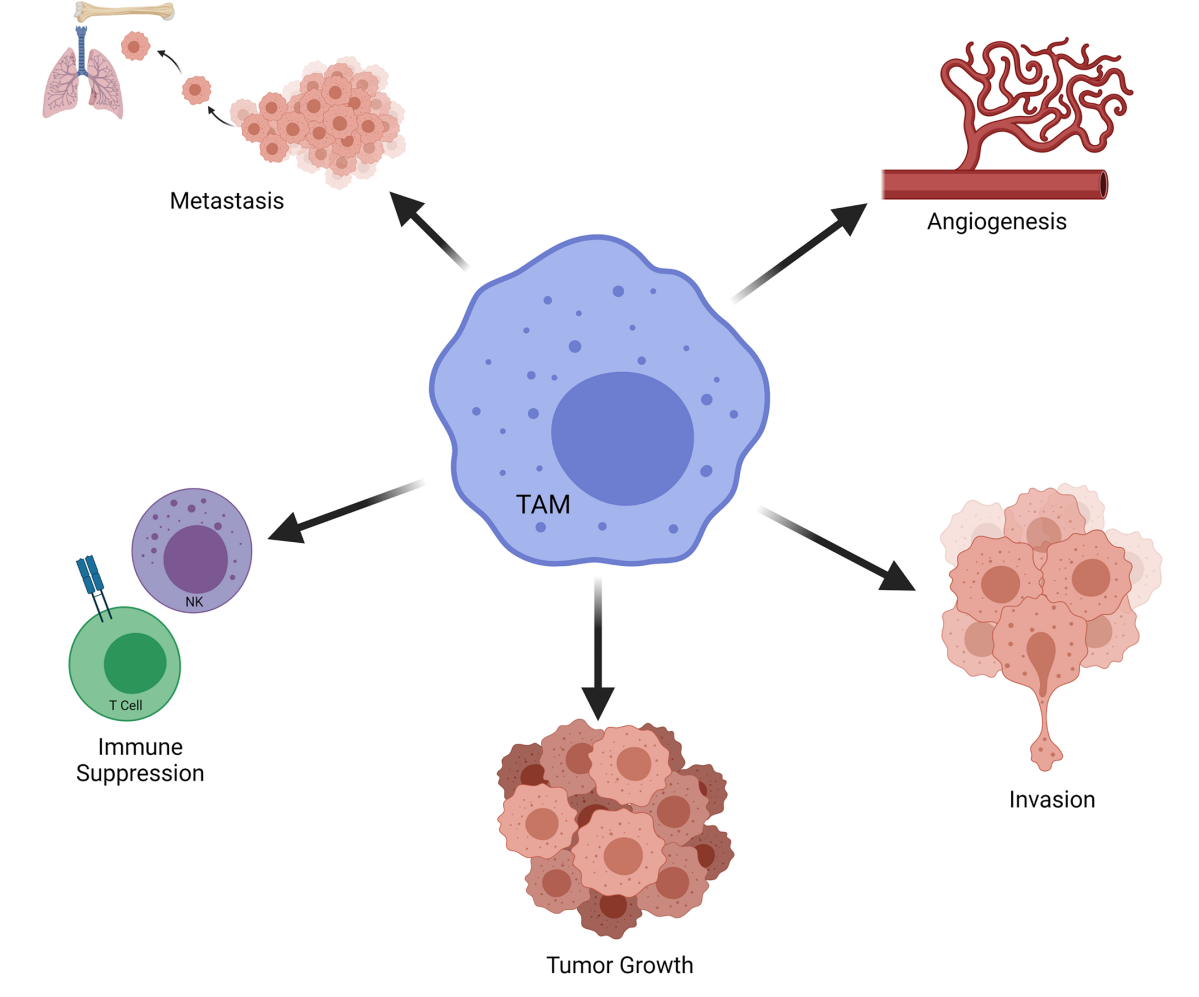
Role of TAMs on Tumor Progression. Selected effects of TAMs in tumorigenesis are demonstrated. NK, natural killer; TAM, tumor associated macrophage.

**Figure 9:**
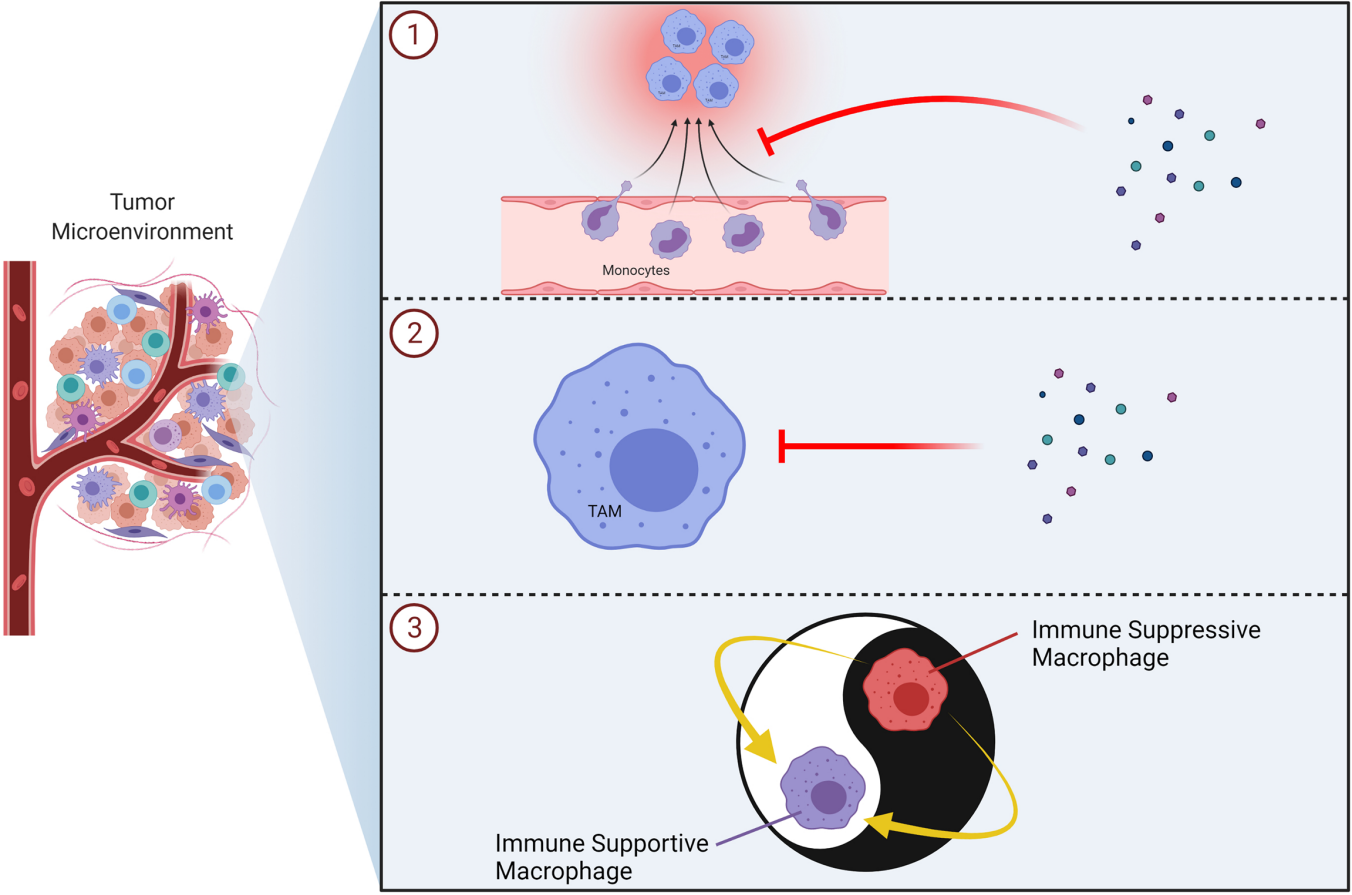
Therapeutic Strategies to Target TAMs in Tumors. Schematic illustrations of the TAM-targeting approaches including inhibition of monocyte/macrophage recruitment (*e.g.* CXCR4, CCR2 inhibitors) ①, inhibition/elimination of macrophages in the tumor microenvironment (*e.g.* bisphosphonates) ② and reprogramming of TAMs into immune supportive macrophages (*e.g.* TLR7/8 agonist) ③ are demonstrated. TAM, tumor associated macrophage; TLR, toll-like receptor.

Macrophages may assume roles in all stages of tumor progression (**Figure 8**) (200, 201). For instance, macrophages are able to induce angiogenesis and help tumor invasion in primary tumor. Subsequently, they may alter the pre-metastatic site, facilitate the dissemination and growth of tumor cells (202). Macrophages mostly favor tumor growth and are associated with poor outcome in various cancers (203). TAMs with an M2 phenotype can promote tumor metastasis and get involved in immune suppression as well as causing failure of radiation or checkpoint inhibitor therapy (204). M2-like TAMs that infiltrate metastatic sites can limit immune responses against high-grade serous carcinomas (205). Metastasis associated macrophages (MAMs) include bone marrow derived macrophages as well as tissue resident macrophages. MAMs can promote metastasis in secondary site (206). Thus, strategies that aim to target macrophages for cancer treatment has been explored over the last decades (**Figure 9**) (203, 207–210). Current strategies to target TAMs for anti-cancer therapy usually involve inhibition/depletion of TAMs by various approaches or reprogramming them to assume an anti-tumor role instead of showing pro-tumoral effects (211–213). M2 macrophages secrete TGF-β, vascular endothelial growth factor (VEGF), IL-6, IL-10 as well as chemokines which facilitate angiogenesis and ECM remodeling. As a result, they can promote tumor invasion and metastasis (214). In addition, macrophages are very effective in creating an immunosuppressive microenvironment. For instance, TAMs may limit the activity of tumor infiltrating lymphocytes (203). Macrophages can stimulate regulatory T cell (T_reg_) development and expansion as well as disrupting natural killer (NK) and T cell functions (200, 210). TAMs may also overexpress inhibitory ligands for T cells such as PD-L1 and programmed cell death 1 ligand 2 (PD-L2) (210). High expression of PD‐L1 by TAMs was reported in hepatocellular carcinoma, glioblastoma and pancreatic cancer (215–218). PD-L1 expressed by antigen-presenting cells (*e.g.* macrophages) is widely regarded as an important regulatory molecule for T cells (219).

Indeed, TAMs constitute a major portion of the tumor microenvironment. Clinical studies suggest that cytokines in the tumor *milieu* influence TAMs, which are inherently plastic cells, to assume an immunosuppressive role (220). It is known that TAMs are usually similar to M2 macrophages (221). Tumor-infiltrating M1 polarized macrophages are usually identified by an IL-12^hi^ IL-10^lo^ phenotype. During tumor development, these macrophages can enhance anti-tumor immune reactions. However, TAMs generally lean towards an M2-like phenotype, which is characterized by an IL-12^lo^ IL-10^hi^ phenotype, at later stages of tumor. Such macrophages demonstrate decreased tumoricidal activity (222). In an interesting study, Pinto et al. profiled macrophages in 150 colorectal cancer cases by immunohistochemistry; utilizing CD68 [as a marker of macrophage lineage (*CD68 is one of good markers of TAMs* (223))], CD80 (as a pro-inflammatory macrophage marker) and CD163 (as an anti-inflammatory macrophage marker). They found that CD163^+^ macrophages were predominantly found at the tumor invasive front, while CD80^+^ ones were almost exclusively located in the adjacent normal mucosa (224). Such findings suggested a chiefly anti-inflammatory polarization of TAMs. Given these findings, it is crucial to identify molecules that affect macrophage plasticity in the tumor *milieu* in order to design macrophage-oriented treatment and diagnosis strategies (225). Although FDA approved cancer immunotherapies primarily target the adaptive immunity, the importance of innate immunity in terms of anti-tumor strategies started to attract a great deal of attention (226). Various approaches have been explored over the recent years in order to target TAMs (227, 228). In line with such strategies, macrophages which express a chimeric antigen receptor (CAR) may prove to be useful in order to target solid tumors (229). In a recent inspiring study, Klichinsky et al. reported transducing human macrophages with an adenovirus vector carrying a CAR (230). They showed that a single infusion of human CAR macrophages decreased tumor burden and prolonged overall survival in two solid tumor xenograft mouse models (230). In another exciting study, Mitragotri et al. recently reported an engineered particle (called as “*backpack*”) which can adhere to the surfaces of macrophage and regulate cellular phenotypes *in vivo*. The “*backpacks*” released cytokines to guide the polarization of macrophages toward anti-tumor phenotypes, slowed tumor growth and improved overall survival in a murine breast cancer model (231). These findings will most likely open new avenues for next-generation CAR applications and adoptive cell transfer strategies in cancer therapy.

## The Interplay Between CAFs and TAMs

In addition to playing important tumor-promoting roles in tumor initiation and progression, CAFs were also shown to sculpt the tumor microenvironment (93, 232). It has been known for some time that CAFs interact with tumor cells by cell-cell contact or *via* release of soluble factors. In addition to such interactions, CAFs also take active part in the crosstalk with other members of the tumor microenvironment. They secrete a plethora of soluble mediators (*e.g.* cytokines, chemokines); which affect various types of leukocytes including macrophages, thereby taking part in immune regulation (70, 197, 233). Moreover, various recent studies have demonstrated that CAFs function as regulators of immune cell recruitment and function (197).

In line with these findings, the effects of stromal fibroblasts on monocytes/macrophages have been investigated by several groups. CAFs may increase monocyte recruitment and differentiation into TAMs (234). CAFs can also promote skin tumor development by maintaining monocyte chemotactic protein-1 (MCP-1) mediated macrophage infiltration and chronic inflammation (235). Podoplanin^+^ CAFs were recently reported to be associated with CD204^+^ TAM infiltration in lung squamous cell carcinoma (236). Moreover, podoplanin^+^ CAFs were also found to be associated with monocyte recruitment and differentiation into CD204^+^ TAMs in lung adenocarcinoma (237). Yavuz et al. demonstrated that CAFs isolated from human invasive breast cancer could facilitate the differentiation of monocytes into M2-like pro-tumoral macrophages, in contrast to normal breast-derived fibroblasts. Such a differentiation was evident in terms of function as well as phenotypic features (15). Furthermore, these CAFs were demonstrated to be puissant in terms of recruiting monocytes. MCP-1 and SDF-1 were shown to be pivotal monocyte chemotactic cytokines which were secreted from stromal cells (15). Human CAFs and M2 macrophages were demonstrated to cooperate during prostate cancer progression (238). Similarly, CAFs and TAMs represent important partakers of tumorigenic processes also in head and neck cancer (239). The interplay between CAFs and TAMs seems to be very complex, as these two groups of cells are also able to alter each other’s functions (23).

### Reciprocal Interactions

It has long been known that tumor cells interact with immune cells in the tumor *milieu*. On the other hand, relatively recent studies have discovered the key effects of the communication of immune cells and stromal cells (*e.g.* CAFs) on tumorigenesis. Stromal cells are able to recruit immune cells in addition to altering their functions. A specific example of such an influence exists in terms of CAFs, which are able to affect monocyte recruitment and polarization. Indeed, CAFs and TAMs are the main components of stromal cells (240–243). Tumor associated macrophages display high level of plasticity. Their activation status in the tumor microenvironment rather has an ephemeral nature (244). There exists a close relation between TAMs and CAFs, as TAMs constitute the most abundant innate immune cell type in the vicinity of CAF populated areas (134). Macrophage derived factors may promote the activation of resident hepatic stellate cells into myofibroblasts, resulting in a fibrotic environment in liver metastases of pancreatic ductal adenocarcinoma (245). Recently, Tokuda et al. showed the significance of osteopontin mediated cancer-TAM-CAF interactions in hepatocellular carcinoma (246).

M2 type TAMs can activate CAFs and thus help tumor progression (247). In addition, M2 macrophages can influence the MMT of fibroblasts, resulting in enhanced reactivity (238). Reciprocally, CAFs are also able to change M1 macrophages in the tumor microenvironment into M2-like macrophages (248). In neuroblastoma, bone marrow-derived MSCs (which become activated into CAFs at tumor site) were found to increase the invasiveness of macrophages; which in turn, induced the proliferation and invasion of CAFs (249). In addition to metabolically supporting the tumor growth, reciprocal interactions of CAFs with M2 macrophages result in a significant pro-tumoral effect (250). Stairs et al. reported that tumor cells recruited immature myeloid cells, which demonstrated immunosuppressive properties and facilitated desmoplasia by activating fibroblasts (251). It should be borne in mind that the reciprocal interactions of CAFs and TAMs in the tumor *milieu* have not been fully discovered, despite the fact that these two cell types are critical constituents of the tumor microenvironment. Thus, novel studies are required to decipher bilateral interactions between CAFs and TAMs as well as to understand the effects of such interactions on diverse functions of both CAFs and TAMs.

In addition to providing mechanistic insights, CAFs and CD163^+^ macrophages (M2) may also prove to be potential prognostic predictors; since expressions of CAF and M2 macrophage markers are correlated with poor prognosis in colorectal cancer and oral squamous cell carcinoma (252, 253). Given the effects of macrophage polarization in terms of anti-tumor immune responses, one can easily foresee that such a trans-differentiation of macrophages from anti-tumoral M1-like to pro-tumoral M2-like macrophages is a pivotal incident in the constitution of the tumor permissive microenvironment.

### Recruitment and Polarization of TAMs by CAFs

It has been demonstrated that most of the macrophages located in tissues originated from yolk sac precursor cells (254, 255). Hence, the notion about adult tissue macrophages being only derived from bone marrow precursor cells has become obsolete. That being said, it should be kept in mind that macrophages found in pathogen associated inflammation chiefly originate from bone marrow monocytes (256). When TAM sub-populations were investigated, most of them were recently demonstrated to emerge from the Ly6C^+^ population of circulating mouse monocytes in grafted tumors, primary mouse mammary tumors and in lung metastases (176, 257, 258).

**Figure 10:**
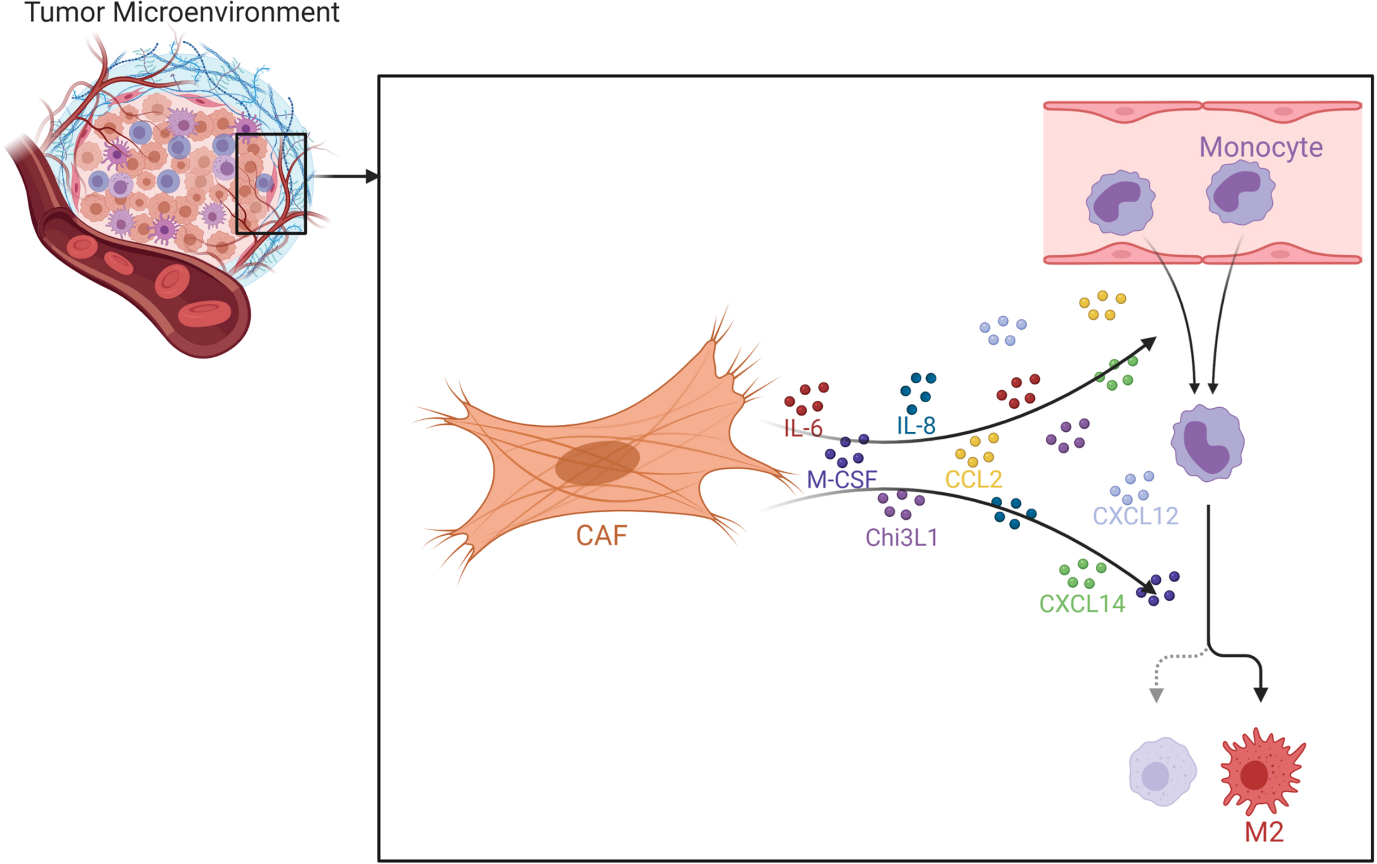
Effects of CAFs on Macrophages. Various chemokines and cytokines secreted by CAFs may promote macrophage recruitment and M2 polarization. CAF, cancer associated fibroblast; Chi3L1, chitinase 3-like 1.

**Figure 11:**
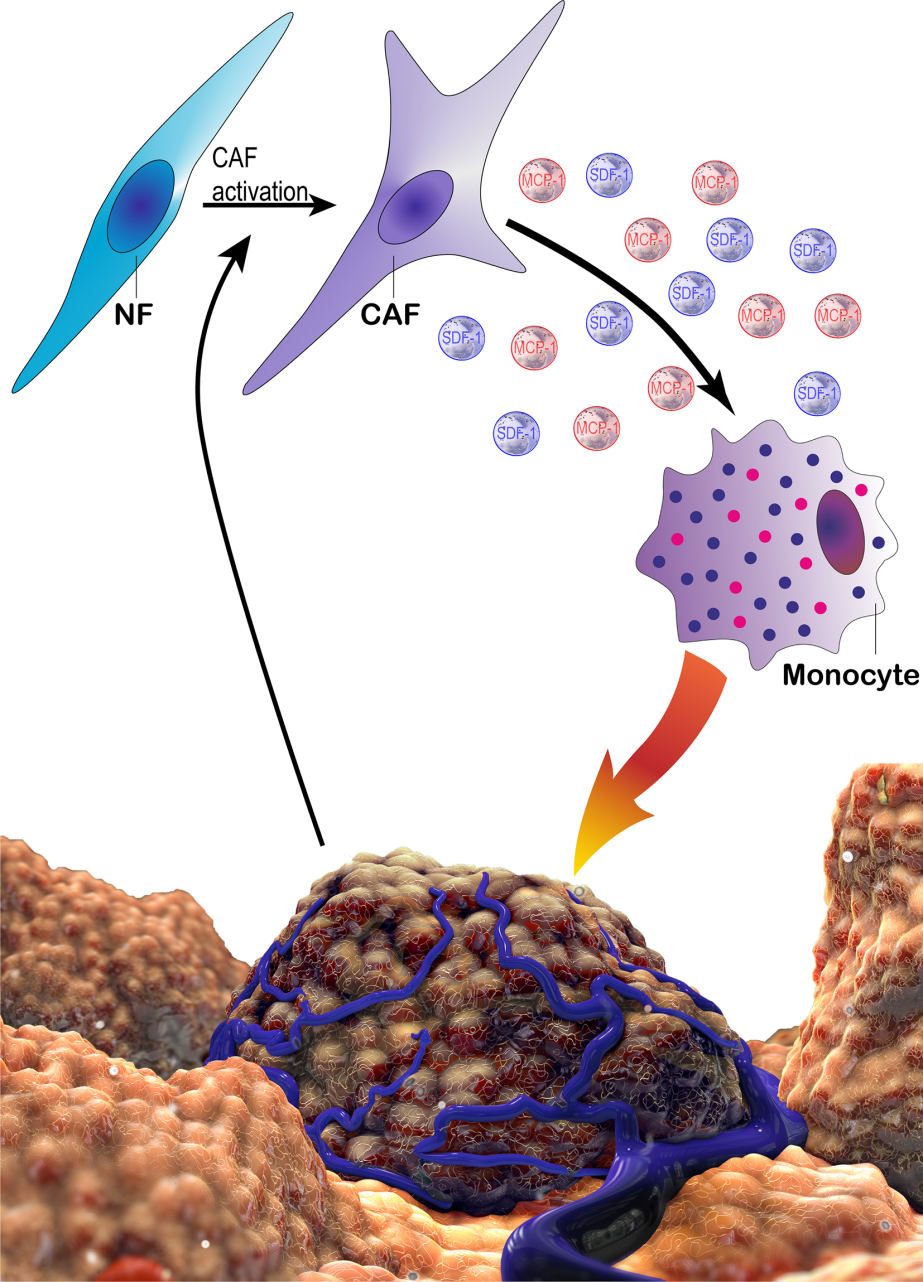
The schematic demonstration of potential tri-directional interactions of cancer cells, CAFs and monocytes. Cancer cells can take part in the activation of CAFs *via* several factors. CAFs may in turn recruit monocytes *via* MCP-1 and SDF-1. In addition, CAFs promote a pro-tumoral phenotype in monocytes/macrophages, which enhance cancer cell proliferation and motility as well as suppressing immune responses. CAF, cancer associated fibroblast; MCP-1, monocyte chemotactic protein-1; NF, normal fibroblast; SDF-1, stromal derived factor 1.

CAFs have been demonstrated to recruit macrophages to the tumor microenvironment in various studies with many mouse models, *e.g.* breast, prostate and squamous cell carcinomas (**Figure 10**) (259–261). CAFs release high amounts of SDF-1, which was demonstrated to be implicated in monocyte recruitment (15). In addition to SDF-1, CAFs are also able to secrete CXCL14, which may promote monocyte recruitment and polarization into M2 macrophages in prostate cancer together with SDF-1 (238, 260). Such findings, in fact, accentuate the pivotal role of SDF-1/CXCR4 axis for the progression of various cancers (262). It is known that MCP-1 can facilitate the infiltration of blood monocytes into CAF spheroids and Ksiazkiewicz et al. demonstrated that MCP-1 is important for the recruitment of blood monocytes to tumor fibroblastic areas (263). Thus, MCP-1 as well as SDF-1 plays a key role in CAF-induced monocyte chemotaxis (**Figure 11**) (15). CAF derived CXCL16 is also able to attract monocytes to promote stroma activation in triple negative breast cancer (264). Mace et al. demonstrated that pancreatic stellate cells, which represent a subset of pancreatic CAFs, produce macrophage colony-stimulating factor (M-CSF), IL-6, VEGF, SDF-1 and MCP-1; which may promote monocyte recruitment and macrophage differentiation as well as potentially facilitating M2 polarization (265, 266). Adenosine generated by CAFs in the tumor microenvironment can induce the expansion and/or differentiation of M2-like macrophages (267–270). Hegab et al. also reported that CAFs increased the conversion of TAMs to the M2 phenotype in a mouse model of lung adenocarcinoma (271). CAF derived IL-6, IL-8, TGF-β, and IL-10 also promote monocyte recruitment and differentiation into M2 macrophages (272, 273). In the human colorectal cancer microenvironment, CAF derived IL-6 and IL-8 were suggested to promote the differentiation of tumor infiltrating myeloid cells into M2 macrophages (274). In an interesting study, Nakamura et al. investigated the significance of expression of carbonic anhydrase IX, which is a marker of hypoxia, by CAFs. They reported that the numbers of CD204^+^ TAMs and podoplanin^+^ CAFs were significantly higher in the carbonic anhydrase IX^+^ CAFs group than in the carbonic anhydrase IX^-^ CAFs group (275).

Higashino et al. reported that human FAP^+^ mesenchymal stem cells (CAF-like cells) helped the growth and migration of peripheral blood mononuclear cell derived macrophage-like cells (276). CAF-like cells promoted M2 polarization of macrophage-like cells. The CAF-like cells secreted CCL2, IL-6, CXCL8; which facilitated the migration of macrophage-like cells. Such effects were found to be associated with FAP expression, since FAP silencing decreased cytokine secretion in CAF-like cells (276). Yang et al. reported that FAP induced inflammatory CAFs by activating STAT3 *via* the uPAR–FAK–c-Src–JAK2 pathway. FAP^+^CAFs were the main source of CCL2, which facilitated the recruitment of myeloid-derived suppressor cells (MDSCs) to the tumor and promoted immunosuppression in a CCR2 dependent manner (277). Indeed, CAFs have a significant effect on polymorphonuclear MDSC infiltration of tumors (278). In another study, Borriello et al. reported isolating a population of αFAP- and FSP-1–expressing CAFs that share functional/phenotypic features with bone marrow derived mesenchymal stromal cells from primary human neuroblastoma tumors (279). In addition, they reported that the presence of αFAP- and FSP-1–positive cells in human neuroblastoma tumor stroma correlated with that of M2 TAMs (279). Similarly, CAFs were also found to be associated with infiltration of CD163^+^ macrophages in triple negative breast cancer and nasopharyngeal carcinoma patients (280, 281). Such studies might provide evidence for the association between CAFs and TAMs as well as their cooperation in generating a pro-tumorigenic *milieu*. Experiments with 3-D cultures of human breast cancer cells and fibroblasts revealed the CCL2-mediated recruitment of monocytes by CAFs (263). CAF derived CXCL12 and CXCL14 were also implicated in macrophage recruitment and M2 polarization (134). In hepatocellular carcinoma, human CAFs can attract monocytes by the SDF-1a/CXCR4 pathway and facilitate their differentiation into MDSCs *via* IL-6-mediated STAT3 activation (282). Zhang et al. reported that human CAFs could promote M2 polarization in pancreatic ductal adenocarcinoma partly *via* M-CSF (283).

In addition to their direct effects on macrophages, CAFs may also indirectly affect macrophage polarization. Pancreatic stellate cells (CAF-like cells) in pancreatic ductal adenocarcinoma were shown to stimulate mast cell activation (284). Mast cells, in turn, produce IL-13 which promotes M2 macrophage polarization (285). Furthermore, human CAF mediated ECM remodeling can increase the infiltration of TAMs, which may show pro-tumorigenic effects (286). Moreover, CAF derived ECM components may modulate macrophage polarization to M2-like (287). Kobayashi et al. demonstrated the impact of stroma derived hyaluronan mediated ECM remodeling on macrophage mobilization. Hyaluronan served as a signal for the recruitment of TAMs and its deficiency impaired macrophage recruitment (288). An interesting study investigated whether radiotherapy influences CAF mediated immunoregulatory effects on macrophages. Ionizing radiation did not alter the immunoregulatory effects of CAFs on macrophages (289). In line with this finding, Gorchs et al. reported that the strong immunosuppressive effect of CAFs on activated T cells remained unchanged after radiotherapy (290). On the other hand, radiotherapy was proposed to increase paracrine signaling between fibroblasts and tumor cells *via* insulin-like growth factor (IGF) and TGF-β pathways (291, 292). Chemotherapy can also modulate the tumor microenvironment (293). CAF derived growth arrest-specific protein 6, which is a ligand of TAM receptors, was reported to increase during cisplatin based chemotherapy (294). Peiris-Pagès et al. reported that tumor cells in contact with fibroblasts could elicit interferon mediated signaling in response to chemotherapy (295). Indeed, chemotherapy may promote the recruitment of monocytic cells and macrophages to tumors *in vivo* (133, 295, 296). In light of these findings, the trilateral crosstalk among TAMs, CAFs and tumor cells merits further investigation in patients treated with radiotherapy or chemotherapy.

Cheng et al. have shown that CAFs may recruit neutrophils and induce PD-L1^+^ neutrophils through the IL-6 - STAT3 pathway which facilitates immune suppression in hepatocellular carcinoma (297). STAT3 is known to affect tumor growth and invasion and the JAK/STAT3 pathway is activated in various cancer types (298–300). CAFs were recently shown to be able to induce the differentiation of recruited monocytes into programmed cell death protein 1 (PD-1) expressing M2-like macrophages, instead of M1 macrophages; in addition to recruiting those monocytes (15). Pathological evaluations of human mammary cancer tissues revealed that the number of CAFs is positively correlated with the numbers of both CD163^+^ and CD206^+^ macrophages. CD206 was demonstrated to be more correlated with M2a-like macrophages, whereas CD163 is noted on the surface of M2c-like macrophages (301, 302). Furthermore, M2a macrophages are known to be related with T_H_2 type immune responses and elimination of parasites. On the other hand, M2c macrophages rather take part in immune regulation and tissue remodeling (303). In light of the current literature, CAFs appear to be inducing the polarization of M1 macrophages more into the M2c phenotype than the M2a. Given the fact that M2c phenotype is associated with immune suppression, such a mechanism would best serve the interests of the tumor instead of anti-tumor immune responses. Moreover, the tissue remodeling roles of M2c macrophages also seem to be cut out for helping tumor progression since tumors are known as wounds which do not heal and; therefore, constitute a chronic wound healing or tissue remodeling process instead of an allergic response or a reaction for elimination of parasites.

In addition to tumor cell and T lymphocyte derived IL-4 (304), CSF-1 and granulocyte-macrophage colony-stimulating factor (GM-CSF), which are secreted from tumor cells (179, 305), were demonstrated to promote pro-tumoral differentiation of macrophages. In congruence with those studies, CAFs are also able to take active part in this re-polarization process. It was reported that the number of TAMs in breast carcinoma is significantly correlated with the presence of CAFs (15). A recent study showed that CAF derived Chitinase 3-like 1, which is implicated in inflammatory disorders, facilitated tumor growth in breast cancer. This effect was also associated with high infiltration of M2 polarized macrophages and T_H_2 type immune responses (261). Enhanced infiltration of breast tumors with TAMs may be caused by the presence of high number of CAFs in such tumor tissues; as CAFs are able to recruit monocytes and promote M2 macrophage polarization.

### The Effects on Tumor Progression

Myriad of studies reported that CAFs and TAMs interact to promote cancer progression (306). In addition, various studies demonstrated that TAMs facilitate breast cancer invasion and metastasis *via* secreting numerous cytokines (307–309). 3-D cultures of human lung adenocarcinoma cells, human lung fibroblast cells and macrophages showed that macrophages and fibroblast cells can facilitate invasion and metastasis (310). CAFs were shown to play key roles in inducing the pro-tumoral phenotype of TAMs (248). Clinicopathological evaluations of oral squamous cell carcinoma tissue samples revealed that both high grade (0: negative, 1: scanty, 2: focal, 3: abundant) of CAFs and high number of CD68^+^ macrophages were correlated with the TNM stage. In addition, high numbers of CD68^+^ or CD163^+^ macrophages were correlated with Ki-67 labeling index. Furthermore, the number of CD68^+^ macrophages influenced progression free survival. Last but not least, the number of M2-polarized macrophages was associated with vascular invasion (248). Miyake et al. demonstrated that high expression of CXCL1 in urothelial cancer of the bladder was associated with increased recruitment of TAMs/CAFs and poor prognosis (311).

The interplay between CAFs and M2 macrophages, collectively promotes cancer cell motility, tumor invasion and metastasis. This collaboration may also activate endothelial cells and result in angiogenesis (238). Furthermore, collaboration of MAMs and CAFs increases the metastatic potential of arriving tumor cells in the secondary site (206). In addition to affecting the behavior of tumor cells and modifying the tumor microenvironment at primary/metastatic sites (125, 312, 313), CAFs can escort tumor cells in the bloodstream and help the tumor cells survive during the migration to metastatic sites (107). Podoplanin^+^ S100A4^+^ PDGF-Rα^+^ fibroblasts were shown to be present in bone metastasis sites of human breast cancer patients, while PDGFRβ^+^ fibroblasts were present in lung metastasis sites (314). Similarly, macrophages were shown to play an important role in tumor recurrence and metastatic outgrowth in murine models (206, 315–319). During the metastatic cascade, CAFs and M2 macrophages can collaborate in altering the functions of other stromal cells as well as the tumor cells (250, 320). Nielsen et al. reported that early recruitment of granulin secreting inflammatory monocytes to the liver is very important for pancreatic ductal adenocarcinoma liver metastasis. Granulin secretion by MAMs activated resident hepatic stellate cells into myofibroblasts that produced periostin, causing a fibrotic *milieu* that supports metastatic tumor growth (245).

The existence of a crosstalk among colon cancer derived exosomes, fibroblast-derived exosomes and macrophage phenotypes in colon cancer metastasis was also proposed (321). In addition, cancer derived exosomes were also shown to be taken up by liver resident Kupffer cells in a mouse model of pancreatic cancer metastasis. The exosomes bore macrophage inhibitory factor that promoted TGF-β generation from Kupffer cells, which activated resident hepatic stellate cells into myofibroblasts. In turn, myofibroblasts prepared the liver for metastatic disseminated tumor cells *via* production of fibronectin to recruit monocytes/macrophages (322–325). In addition, it was also reported that the primary tumor promoted fibroblast secretion of fibronectin, which took part in the recruitment of bone marrow derived macrophages to the secondary site in liver and lung metastases (322, 324, 326, 327). In a recent study, CAFs and TAMs were reported to interact in generating a perlecan (a heparan sulfate proteoglycan that stores and stabilizes growth factors implicated in regulation of prostate cancer cell growth) rich desmoplastic stroma at sites of prostate cancer bone metastasis (328). Indeed, the complex trilateral crosstalk among cancer cells, CAFs and M2 macrophages may activate endothelial cells and their bone marrow derived precursors to induce *de novo* angiogenesis and promote metastatic spread of tumor cells (238). CAFs can actively recruit endothelial progenitor cells to the tumor site. Endothelial progenitor cells synergize with CAFs to promote epigenetic modifications of tumor cells *via* mesenchymal-to-amoeboid transition, which provides an advantage to metastatic cells (329). In fact, Duda et al. demonstrated that metastatic cells can bring stromal components (*e.g.* activated fibroblasts) from the primary site to the lungs (107). The viability of circulating metastatic cancer cells was found to be higher if they were incorporated in heterotypic tumor stroma cell fragments. In addition, those stromal cells could provide a growth advantage to the metastatic tumor cells. Indeed, circulating tumor cells (CTCs) do not act independently and they are assisted by stromal and immune cells, which alter their metastatic potential. CAFs, which were reported to be associated with CTCs in heterotypic CTC clusters, seem to increase the metastatic efficiency (330). Interestingly, CAFs were found to be present in the brain metastases from lung carcinoma and other carcinomas, in contrast to primary brain tumors or normal brain tissue (107). Such findings may prove the direct involvement of primary tumor stroma in metastasis.

M2 macrophages which were induced by CAFs were demonstrated to promote pancreatic tumor cell growth, migration and invasion (283). In another study, CCR2-dependent recruitment of monocytes/macrophages by tumor resident mesenchymal stromal cells was reported to facilitate tumor growth (331). A recent study showed that endosialin-positive CAFs recruited macrophages and promoted the M2 polarization; thus, promoting the progression of hepatocellular cancer (332). Hashimoto et al. suggested that TAMs and CAFs collectively facilitate the development of neuroblastoma (249). Myofibroblasts are known to secrete VEGF, which induces the accumulation of immune cells such as macrophages at sites of fibrosis (333). In turn, VEGF mediated activation of macrophages promotes skin cancer carcinogenesis, angiogenesis and invasion (334).

CAF-educated monocytes were demonstrated to enhance breast cancer cell invasion; to significantly increase the expressions of EMT-related genes and vimentin protein; while decreasing the expression of E-cadherin protein; in contrast to their normal counterparts (15). In addition, CAF-educated monocytes increased the motility of breast cancer cells. Linde et al. recently reported that CD206^+^ macrophages downregulated E-cadherin junctions in breast cancer cells (335), which in turn may promote EMT. In light of these findings, CAFs seem to facilitate EMT, invasion and metastasis (100); through promoting M2-polarized macrophages. It was also reported that CAF-educated monocytes increased breast cancer cell proliferation *in vitro* (15). Furthermore, the infiltration of CD163^+^ TAMs seemed to be associated with specific molecular subtypes of breast cancer; since their numbers were found to be the highest in triple negative, and the lowest in ER^+^PR^+^ breast cancer samples. Recent findings suggest that the tumor stroma also participates in the tumor resistance to therapy (23, 336). Ireland et al. demonstrated that TAMs and CAFs are major sources of IGF-1 and IGF-2 in breast and pancreatic tumors; and IGF signaling is implicated in the breast and pancreatic tumor resistance to paclitaxel and gemcitabine, respectively (337, 338).

### The Effects on Immune Evasion

CAFs may be implicated in the establishment of an immunosuppressive *milieu via* inducing immunosuppressive macrophages. Takahashi et al. analyzed the effects of CAFs on functional polarization of TAMs in oral squamous cell carcinoma and found that infiltration of CAFs was associated with the numbers of CD68^+^ CD163^+^ macrophages. In addition, they demonstrated that CAFs promoted the accumulation of pro-tumoral macrophages, resulting in an immunosuppressive *milieu* (248). CAF-supernatant-treated CD14^+^ cells (CAF-educated cells) resembled pro-tumoral macrophages and these CAF-educated cells significantly suppressed both CD4^+^ and CD8^+^ T cell proliferations. Moreover, the suppression of T cell proliferation was mediated *via* the production of TGF-β, IL-10, and arginase I (248). CAF derived IL-33 was reported to cause TAMs to undergo the M1 to M2 transition (339). Genomic profiling of metastasis related genes revealed that these IL-33 stimulated TAMs demonstrated a significant increase in MMP9 expression. Moreover, genetic deletion of IL-33 or MMP9 blocked metastasis in mouse and human fibroblast rich pancreatic cancers (339). Indeed, these results may shed light on the TAM-CAF committed cancer metastasis as well as underlining the importance of targeting the TAM-CAF axis in terms of a potential cancer treatment. In a recent article, Shani et al. demonstrated that IL-33 was upregulated in metastasis associated fibroblasts in mouse models of spontaneous breast cancer metastasis and in patients with breast cancer with lung metastasis (340). IL-33 upregulation promoted type 2 inflammation in the metastatic microenvironment and took part in the recruitment of various cells including inflammatory monocytes to lung metastases. *in vivo* IL-33 targeting caused inhibition of lung metastasis and significant reduction of immune cell recruitment (340). In a recent study, Sun et al. reported that CXCL3 was highly upregulated in IL-33-stimulated macrophages and the receptor of CXCL3 (CXCR2) was mostly expressed by CAFs (341). In addition, CXCL3-CXCR2 signaling upregulated α-SMA and activation of CXCR2 by CXCL3 caused CAF-to-myoCAF transition (341). These findings underline the importance of the interaction between CAFs and immune cells in terms of generating a favorable inflammatory niche in breast cancer lung metastasis.

Adenosine, which is an immunosuppressive metabolite produced at high levels in the tumor microenvironment, is implicated in immune escape as well as in tumor growth and metastasis (342). Various cell types can generate adenosine in the tumor microenvironment such as CAFs as well as tumor cells (267, 268, 343–345). Adenosine can exert immunosuppressive effects on/via several immune cells including T cells, NK cells, dendritic cells (DCs) and MDSCs as well as macrophages (268, 346). In the extracellular space, adenosine exerts a plethora of immunomodulatory effects. It alters mononuclear phagocyte functions *via* 4 G-protein coupled cell membrane receptors (A_1_, A_2A_, A_2B_, and A_3_) (347). Adenosine downregulates classical macrophage activation majorly *via* A_2A_ receptors, while upregulating alternative macrophage activation *via* A_2B_ receptors (347). Hence, adenosine affects the process of tumorigenesis by regulating mononuclear phagocyte functions and extracellular adenosine generation is one of various immunosuppressive mechanisms that hinder anti-tumor immune responses in part due to the interactions between CAFs and TAMs (268).

Moreover, the interplay between these two cells types may also demonstrate critical effects on adaptive immune responses at various levels. One of such immunomodulatory effects on adaptive immunity is associated with the disruption of antigen presentation. CAF derived TGF-β, IL-6, tryptophan 2,3-dioxygenase (TDO2), indoleamine-2,3-dioxygenase (IDO) and VEGF can impede the function and maturation of DCs; resulting in the inhibition of T cell activation and causing T cell anergy (134, 234, 348–352). IL-6 signaling may also redirect monocyte differentiation into macrophages rather than DCs (24, 134, 353). In addition, Yavuz et al. demonstrated that CAF-educated monocytes significantly suppressed T cell proliferation in contrast to control monocytes (15). CAFs were previously reported to directly inhibit T lymphocyte proliferation (232, 354). In addition to their direct effects, CAFs also indirectly suppress immune responses *via* polarizing the monocytes to M2-like TAMs. Furthermore, CAF-educated M1 macrophages were also demonstrated to increase secretion of IL-10 and decrease secretion of IL-12 in addition to increasing the expression of M2 markers (15). Moreover, CAFs can induce a PD-1^+^ TAM phenotype by themselves, even without the presence of cancer cells (15). Nearly all PD-1^+^ TAMs express an M2-like surface profile (355). Thus, PD-1 axis may be pivotal in CAF mediated immune suppression *in vivo* in various types of cancer. Furthermore, CAF-educated monocytes demonstrated enhanced expressions of CD206 and CD163 (15). Given the fact that CD206 has anti-inflammatory effects, CAF mediated induction of higher expression of CD206 on monocytes might also represent another indirect mechanism of immune suppression by CAFs. Moreover, CAFs were shown to induce down regulation of major histocompatibility complex (MHC) class II expression on monocytes. This finding might demonstrate a mechanism of immune escape through decreased antigen presentation, in addition to confirming that CAFs are not implicated in any types of “good inflammatory response” (356).

CAFs and TAMs were also reported to localize in colorectal carcinomas where they promote the progression of tumor and suppress immune responses. Zhang et al. demonstrated that the recruitment of TAMs was associated with vascular cell adhesion molecule 1 (VCAM-1) expression (272). CAFs are able to induce monocyte adhesion by up-regulating VCAM-1 expression in cancer cells. In addition, CAFs are also able to promote the polarization to M2 macrophages, which take part in suppression of NK cell functions. Thus, CAF-TAM interplay in the tumor microenvironment seems to also have the potential to regulate NK cell functions (272).

## Discussion and Concluding Remarks

Several issues still persist in terms of the effects of CAFs on tumor progression. One of the most important problems that needs to be solved is indeed the origins of CAFs in different cancer types. Given the heterogeneity of CAFs, subpopulations of these cells should also be thoroughly investigated in terms of their specific effects on tumor progression, immune suppression and tumor microenvironment organization. In fact, presence of different fibroblast subpopulations and the effect of the cancer type probably influence the net outcome (135). A solution for the issues concerning the origins and heterogeneity of CAFs could be profiling CAF populations at the single cell level in different cancer settings as well as utilizing multiplex immunofluorescence or brightfield immunohistochemistry (357–360). Indeed, Muhl et al. compared single-cell transcriptional profiles of fibroblasts among murine organs (*i.e.* heart, skeletal muscle, intestine and bladder) in a recent study in order to investigate the heterogeneity of fibroblasts within and between organs (21). As one would expect, they reported significant inter- and intra-organ heterogeneity amongst fibroblasts. In another study, Bartoschek et al. reported functionally and spatially distinct subclasses of CAFs by single cell RNA sequencing in a genetically engineered mouse model (38). They proposed that spatial separation of the CAF subclasses could be attributed to different origins. Such studies will help us understand the relevance of fibroblast heterogeneity in different organs and tissues. Novel studies which will focus on CAFs in addition to fibroblasts, especially in human tissues, will most likely shed new light on the importance of these quirky cells. Moreover, longitudinal studies are required in order to decipher the functions and heterogeneity of CAFs over time, yielding spatiotemporal resolution. Moreover, investigation of primary and metastatic tumors at single cell resolution could help in understanding the role of local and recruited stromal cell populations in carcinogenesis.

The current literature suggests that the collaboration of CAFs with TAMs plays a crucial role in tumor progression. It seems clear that CAFs and TAMs are pivotal components of the tumor microenvironment and they may prove to be potential valuable therapeutic targets. Given the reciprocal relationship of CAFs with TAMs in the tumor microenvironment in terms of promoting tumor growth, therapeutic strategies aimed at altering polarization of TAMs or reeducating M2 macrophages should also take into account the synergistic effects of CAFs in order to stimulate effective anti-tumor immune responses. Hence, combined targeting of CAFs and TAMs can be regarded as an option to consider. A better understanding of the mechanisms as well as signaling pathways implicated in the tripartite interplay of CAFs, TAMs and tumor cells will help decipher key concepts of tumor immunology. The fact that there are significant differences among monocytes/macrophages from distinct tumors and the existence of diverse TAM subsets should also be borne in mind (361). Utilization of novel techniques (*e.g.* multiplexed immunohistochemistry, mass cytometry by time-of-flight, single-cell RNA-seq, spatial transcriptomics and systems biology approaches) may indeed assist in better understanding the diversity of TAMs (228, 361).

Future *in vivo* studies will further provide us with novel mechanistic insights and distinct implications. Developing model systems which better simulate all the elements of the tumor microenvironment is crucial in order to reach such goals. These systems will potentially allow for the demonstration of the effects of cell-to-cell contact in addition to the cross-talk mediated though soluble factors. An approach utilizing a severe combined immunodeficiency (SCID) mice setting might help to better determine whether human monocytes co-injected *s.c.* with cancer cells display a potent pro-tumoral effect if preconditioned with CAFs in comparison to their normal counterparts. Given the recent exciting findings concerning the utilization of TAMs for cancer therapy (362–364), harnessing the power of innate immune cells (*e.g. via* CAR macrophages) holds great promise for the development of novel and attractive immunotherapeutic modalities. It is evident that CAFs are able to sculpt the tumor microenvironment *via* TAMs. Therefore, therapeutic strategies which antagonize the CAF-mediated immunosuppressive microenvironment may prove to be efficient in terms of increasing the effectiveness of conventional therapies and immunotherapies against cancer. In addition, a more thorough understanding of the tri-directional interactions of CAFs, TAMs and cancer cells in terms of tumor progression will pave the way for the identification of novel theranostic cues in order to better target the crucial mechanisms of carcinogenesis.

## Author Contributions

The author confirms being the sole contributor of this work and has approved it for publication. **Figures 1**, **3**–**10** were created with BioRender.com.

## Conflict of Interest

The author declares that the research was conducted in the absence of any commercial or financial relationships that could be construed as a potential conflict of interest.

## Glossary

CAF: Cancer associated fibroblast
CAR: Chimeric antigen receptor
CCL: C-C motif chemokine ligand
CCR: C-C motif chemokine receptor
CD: Cluster of differentiation
CDKN1B: Cyclin dependent kinase inhibitor 1B
Chi3L1: Chitinase 3-like 1
CSF-1: Colony stimulating factor 1
CTCs: Circulating tumor cells
CTLA-4: Cytotoxic T-lymphocyte associated protein 4
CXCL: C-X-C motif chemokine ligand
CXCR: C-X-C motif chemokine receptor
DC: Dendritic cell
ECM: Extracellular matrix
EGF: Epidermal growth factor
EGFR: Epidermal growth factor receptor
EMT: Epithelial-mesenchymal transition
EndMT: Endothelial to mesenchymal transition
ER: Estrogen receptor
FAP: Fibroblast activation protein
FGF: Fibroblast growth factor
FSP1: Fibroblast-specific protein 1
GM-CSF: Granulocyte-macrophage colony-stimulating factor
GPR77: G protein-coupled receptor 77
HGF: Hepatocyte growth factor
HLA: Human leukocyte antigen
IDO: Indoleamine-2,3-dioxygenase
IFNγ: Interferon gamma
IGF: Insulin-like growth factor
IL: Interleukin
ING5: Inhibitor of growth family member 5
iPSCs: Induced pluripotent stem cells
LPS: Lipopolysaccharide
MAM: Metastasis associated macrophage
MCP-1: Monocyte chemotactic protein-1
M-CSF: Macrophage colony-stimulating factor
MDSC: Myeloid-derived suppressor cell
MHC: Major histocompatibility complex
MMT: Mesenchymal–mesenchymal transition
Mrc1: Mannose receptor C-type 1
MSC: Mesenchymal stem cell
NF: Normal fibroblast
NG2: Neuron-glial antigen-2
NK: Natural killer
PD-1: Programmed cell death protein 1
PDGF: Platelet-derived growth factor
PDGFR: Platelet-derived growth factor receptor
PD-L1: Programmed cell death 1 ligand 1
PD-L2: Programmed cell death 1 ligand 2
PR: Progesterone receptor
SCID: Severe combined immunodeficiency
SDF-1: Stromal derived factor 1
SMA: Smooth muscle actin
STAT3: Signal transducer and activator of transcription 3
TAMs: Tumor associated macrophage
TCGA: The Cancer Genome Atlas
TDO2: Tryptophan 2,3-dioxygenase
TGF-β: Transforming growth factor-β;
T_H_: T helper cell
TLR: Toll-like receptor
TNF: Tumor necrosis factor
T_reg_: Regulatory T cell
VCAM-1: Vascular cell adhesion molecule 1
VEGF: Vascular endothelial growth factor
